# Genetic Characterization of Swine Influenza Viruses in Thailand in 2019–2025 Reveals Novel Reassortants

**DOI:** 10.1155/tbed/9516354

**Published:** 2026-06-02

**Authors:** Supassama Chaiyawong, Hnin Wai Phyu, Chanakarn Nasamran, Supanat Boonyapisitsopa, Napawan Bunpapong, Somsak Pakpinyo, Kamonpan Charoenkul, Alongkorn Amonsin

**Affiliations:** ^1^ Department of Veterinary Public Health, Faculty of Veterinary Science, Chulalongkorn University, Bangkok, 10330, Thailand, chula.ac.th; ^2^ Emerging and Re-emerging Infectious Diseases in Animals, Center of Excellence, Faculty of Veterinary Science, Chulalongkorn University, Bangkok, 10330, Thailand, chula.ac.th; ^3^ Veterinary Diagnostic Laboratory, Faculty of Veterinary Science, Chulalongkorn University, Bangkok, 10330, Thailand, chula.ac.th

**Keywords:** genetic diversity, reassortant, swine influenza, Thailand

## Abstract

Swine influenza viruses (swIAVs) pose a significant zoonotic threat. Ongoing surveillance of swIAVs in Thailand is essential due to intensive pig farming, which promotes interspecies transmission and the emergence of novel or virulent viruses. This study aimed to conduct a multiyear survey to determine the prevalence and genetically characterize the swIAVs circulating in Thai pig populations from 2019 to 2025. A total of 1629 nasal swab samples from 21 pig farms across 11 provinces in Thailand were collected and tested for swIAV. Our results showed that 10.87% (177/1629) of the samples tested positive for swIAV using *M* gene‐specific real‐time RT‐PCR. Of the 177 positive samples, we successfully isolated 30 swIAVs, which were subtyped as swIAV‐H1N1 (*n* = 14), swIAV‐H1N2 (*n* = 3), and swIAV‐H3N2 (*n* = 13). All 30 swIAVs were subsequently characterized by whole‐genome sequencing, which identified eight swIAV genotypes. Phylogenetic analysis confirmed reassortment among all subtypes (H1N1, H1N2, and H3N2). Most genes across all subtypes originated from the pandemic H1N1‐2009 (pdm) lineage, frequently reassorted with classical swine (CS), Eurasian Avian (EA), or human seasonal (HU) segments. Importantly, four new swIAV genotypes have not been reported in Thailand: endemic H1N1 (8EA), endemic H3N2 (6EA + 2HU), reassortant H1N1 (5pdm + 2EA + 1CS), and reassortant H3N2 (4pdm + 2CS + 2HU). Our results reinforced that pdmH1N1‐2009 was predominantly circulating or was introduced multiple times in pig farms, leading to various reassortments and diverse swIAV genotypes. Since endemic swIAV, pdmH1N1‐2009, and reassorted swIAVs are circulating within the pig population in Thailand, it is important to emphasize the routine monitoring of influenza viruses among pigs, farm workers, and veterinarians on pig farms. Ongoing improvements in surveillance are essential to prevent the emergence of genetically complex swIAVs with pandemic potential.

## 1. Introduction

Swine influenza, caused by the influenza A virus (IAV), is a respiratory disease that affects pigs and poses a significant zoonotic threat globally. Swine influenza viruses (swIAVs) of subtypes H1N1, H3N2, and H1N2 are widely distributed and circulate in domestic pig populations worldwide. The role of pigs as a biological “mixing vessel” is important to influenza ecology because swine respiratory epithelial cells contain receptors for both avian IAVs (alpha‐2,3‐linked sialic acids) and human IAVs (alpha‐2,6‐linked sialic acids). Thus, pigs can serve as hosts for genetic reassortment, leading to the emergence of novel, potentially pandemic influenza viruses [1, 2].

The first swine‐like H1N1 (swIAV‐H1N1) was identified in humans during the 1918 influenza pandemic. The pandemic H1N1‐1918 of avian origin jumped to humans, causing the 1918 pandemic, and then from humans to swine, where it became established as swIAV‐H1N1. It was isolated in North America in 1930 and was called “Classical swine influenza virus; CS‐H1N1” [3, 4]. CS‐H1N1 was predominant in North America and then spread to Europe and Asia. In the 1970s, the classical swine (CS) influenza virus was replaced by an avian‐like swIAV‐H1N1, which was called the “Eurasian avian‐like swine influenza H1N1 virus (EA‐H1N1)” [5, 6].

In the 1990s, triple reassortant (TR) H3N2 and H1N2 emerged in North American swine. A TR H3N2 contained genes from human (*HA, NA*, and *PB1*), swine (NS, NP, and M), and avian (PB2 and PA) viruses [7]. While the emergence of swIAV‐H1N2 was a result of the reassortment between TR H3N2 and classical H1N1 [8]. In 2009, the pandemic H1N1‐2009 (pdm H1N1‐2009) emerged, causing the recent influenza pandemic. The virus was a reassortant with EA‐H1N1 (*NA* and *M* genes), *triple-reassorted internal genes* (*TRIG*; PB2, PB1, PA, NP, and NS), and CS‐H1N1 (HA) [9].

In Thailand, the pig industry is a major livestock industry. The pig population is estimated to be 11.72 million in 2025. It is noted that the Thai pig population decreased by around 18% since 2021 due to the outbreak of African swine fever (ASF) [10, 11]. Pig density in Thailand is relatively high in the eastern, western, and central parts of the country (Supporting Information 1: Figure S1). Pig farm sizes can be categorized into large‐scale commercial farms (>5000 pigs), medium‐scale commercial farms (500–5000 pigs), small‐scale commercial farms (50–500 pigs), commercial smallholder farms (5–50 pigs), and backyard farms (<5 pigs) [12]. Pig housing systems are mostly conventional stables (open‐air concrete pens) and a small number of modern pig farms that use evaporative cooling systems (EVAP). Most of the live pigs (breeders) were imported from Europe (e.g., Denmark) and North America (e.g., Canada). It should be noted that swine influenza vaccination is not routinely practiced in Thai pig farms [13].

In Southeast Asia, Thailand is particularly significant for swIAV ecology due to its dense pig population and intense livestock‐human interface. The presence of swIAVs in Thailand has been documented, with swIAV‐H3N2, H1N1, and H1N2 subtypes, which were first reported in 1978, 1988, and 2005, respectively [14–16]. Prior to the 2009 influenza pandemic (pdmH1N1−2009), the Thai endemic swIAV‐H1N1 (enH1N1) typically comprised two distinct genotypes enH1N1 (7 + 1) and enH1N1 (6 + 2). The enH1N1 (7 + 1) was composed of seven genes from the Eurasian Avian (EA)‐like swine lineage and one gene (*HA*) from the CS lineage, while the enH1N1 (6 + 2) was composed of six genes from the EA and two genes (*HA* and *NS*) from the CS [17]. However, the genetic patterns of Thai endemic swIAV‐H3N2 (enH3N2) revealed various combinations of the EA, CS, and human seasonal (HU) lineages, reflecting the genetic diversity of the enH3N2 ecosystem [18, 19].

The introduction of the pdmH1N1‐2009 virus into the swine population was important for the evolution of swIAV. pdmH1N1‐2009 became established in domestic pigs, leading to endemic swIAVs that reassorted with pdmH1N1‐2009. Many studies have indicated that reassortant swIAVs (r‐swIAVs) with the pdmH1N1‐2009 backbone have emerged as the predominant swIAVs in pigs in Thailand and several other countries. This reassortment, often involving the internal genes of pdmH1N1‐2009 and the surface genes of preexisting endemic swIAVs, presents continuous antigenic shift and drift, complicating regional disease control and surveillance efforts [2, 20]. Zoonotic and reverse zoonotic transmission between humans and pigs has been documented as pig farm owners and workers often come into contact with pigs. For example, a previous report documented swIAV isolated from a 4‐year‐old child in Thailand [21]. Moreover, after the H1N1‐2009 pandemic, there was a report of human‐to‐pig transmission in Thailand in December 2009 [17, 22]. It has also been reported that zoonotic transmission of swIAV to humans has occurred in several countries [23–26].

This ongoing and extensive genetic exchange has significant implications for public health, particularly with the increase in reassorted swIAV variants worldwide. For instance, surveillance in China identified a novel reassorted swIAV‐H1N1, designated genotype G4 (H1N1‐G4), which demonstrated increased prevalence in pigs and enhanced potential for zoonotic spillover [27, 28]. This H1N1‐G4 comprised internal protein genes (*PB2*, *PB1*, *PA, NP*, and *M*) derived from the pdmH1N1−2009, one gene (*NS*) from its TR ancestor, and two surface protein genes (*HA* and *NA*) from the EA. The emergence of H1N1‐G4, which was genetically adapted to swine, reinforced the need for continuous, in‐depth surveillance in high‐risk areas, particularly in Southeast Asia, where environmental conditions and farming practices increased the risk of interspecies transmission [29].

Despite the evidence of rapid swIAV evolution, genomic surveillance following the establishment of the pdmH1N1‐2009 lineage remained limited for the swine population in Thailand. Therefore, this study aimed to conduct a multiyear survey of swIAVs on pig farms across Thailand from 2019 to 2025. The study objectives were to determine the prevalence of Thai swIAVs, to genetically characterize swIAVs through whole‐genome sequencing, and to analyze genetic patterns, diversity, and reassortment dynamics. Through this comprehensive analysis, we identified four new swIAV genotypes not previously reported in Thailand, providing crucial, up‐to‐date insights into the regional swIAV perspective and its potential for zoonotic emergence.

## 2. Materials and Methods

### 2.1. Sample Collection

From 2019 to 2025, we conducted a multiyear swIAV active surveillance program across 21 pig farms in 11 provinces of Thailand. The pig farms are located in Chainat (*n* = 2), Chiangmai (*n* = 1), Chonburi (*n* = 4), Lopburi (*n* = 1), Nakhon Pathom (*n* = 3), Nakhon Ratchasima (*n* = 2), Phetchaburi (*n* = 1), Prachinburi (*n* = 1), Ratchaburi (*n* = 4), Saraburi (*n* = 1), and Suphanburi (*n* = 1) provinces. The pig farms were selected based on prior history of swine influenza outbreaks, cooperation from the farm owners, and convenience for travel and sample transport. A total of *n* = 1629 nasal swab samples were collected from pigs across four age groups: suckling pigs (≤4 weeks) (*n* = 161), nursery pigs (>4–8 weeks) (*n* = 898), fattening pigs (>8–20 weeks) (*n* = 367), and breeder pigs (gilt, sow, and boar) (*n* = 203). Nasal swab samples were randomly collected from pigs with and without respiratory signs using flocked nylon swabs and placed into 1–2 mL of Viral Transport Media (VTM) (minimum essential medium [MEM] with 7% bovine serum albumin [BSA], 100 U/mL penicillin, 100 mg/mL streptomycin, and 1 mg/mL trypsin). Samples were transported at 4°C to the laboratory and stored at −80°C until further use. Detailed animal and pig farm data, including age, farm health status, farm location, and type of pig farm, were recorded during the sample collection. Sample collection was conducted in accordance with the Chulalongkorn University Animal Care and Use Committee (CU‐ACUC) protocol number CU‐VET IACUC# 2131033. Prior to sample collection, verbal informed consent was obtained from all participating farm owners.

### 2.2. swIAV Detection

Viral RNA was extracted from all nasal swab samples using the GeneAll GENTi Viral DNA/RNA Extraction Kit (GeneAll; Lisbon, Portugal) on a Genti Automated Extraction System (GeneAll; Lisbon, Portugal). In brief, 200 µL of the swab sample was added to the extraction plate with 7 µL of the RNA carrier. The RNA sample was extracted using the automated extraction system machine under the viral extraction program. All RNA samples were tested for IAV using one‐step real‐time RT‐PCR. IAV detection was performed using a one‐step real‐time RT‐PCR with the SuperScript III Platinum One‐Step Quantitative RT‐PCR System (Invitrogen; California, USA) with primers and probes specific to the *M* gene [30]. In detail, the 25 µL reagent mixture contained 1x master mix buffer, 0.6 mM MgSO_4_, 1 unit of Superscript III reverse transcriptase, 0.8 µM of forward and reverse primers specific for the *M* gene, 0.2 µM of probe, RNase‐free water, and 4 µL of RNA sample. The one‐step real‐time RT‐PCR protocol consists of the following steps: (1) reverse transcription at 50°C for 30 min, (2) predenaturation at 95°C for 15 min, and (3) 45 cycles of denaturation at 95°C for 15 s followed by annealing‐extension at 60°C for 30 s. Real‐time RT‐PCR results were interpreted using the cycle threshold (Ct value): samples with a Ct value of <36 were considered positive, while samples with a Ct value between 36 and 40 were considered suspects.

### 2.3. swIAV Isolation and Propagation

All positive and suspect samples were subjected to virus isolation and propagation using egg inoculation and/or cell culture, following the World Organisation for Animal Health (WOAH) guidelines [31]. For virus isolation by egg inoculation, the VTM containing the swab samples was vortexed and centrifuged at 2000 g for 20 s. About 100 µL of the supernatant was inoculated into three 9–11‐day‐old embryonated chicken eggs, which were then incubated at 37°C for 5 days. The allantoic fluid from each egg was harvested and tested for hemagglutinin activity (HA) using a hemagglutination test (HA test) with a 0.5% suspension of turkey red blood cells. The HA test was performed using serial twofold dilutions of allantoic fluid, and RBC agglutination was observed after 1 h. The positive allantoic fluid demonstrated HA with a titer ≥2 HA units. For virus isolation using cell culture, 100 μL of the nasal swab supernatant was inoculated onto Madin‐Darby Canine Kidney (MDCK) cells in a culture plate. The cell cultures were incubated at 37°C for 48 h. Cytopathic effects (CPEs) were observed daily during the 48 h of incubation. Supernatants from CPE‐positive samples were subsequently subjected to the HA test [31].

### 2.4. swIAV Genetic Characterization

Whole‐genome sequencing was performed on 30 Thai swIAVs using Oxford Nanopore Technologies. The eight viral gene segments were amplified via conventional one‐step RT‐PCR using the SuperScript III RT‐PCR System with Platinum Taq DNA polymerase (Invitrogen, California, USA) and the universal influenza primers MBT12 and MBT13 [32]. The RT‐PCR products were purified, and a library was prepared using the Oxford Nanopore Rapid Sequencing Kit V14 (SQK‐RAD114; Oxford Nanopore Technologies, Oxford, UK). The library preparation involved mixing 5 µL of the purified PCR product with 0.5 µL of fragmentation mix (FRA), followed by incubation at 30°C for 2 min and then at 80°C for 2 min. Subsequently, 0.5 µL of diluted rapid adapter (RA) was added, followed by incubation at 25°C for 5 min. The final sequencing mix was completed by adding 15 µL of sequencing buffer (SB) and 10 µL of library beads (LIBs). Sequencing was performed on a Flongle flow cell R10 version (Oxford Nanopore Technologies, Oxford, UK). The flow cell was primed with a priming mix (117 µL of flush buffer (FB) and 3 µL of flush tether [FLT]) before the sequencing mix was loaded. Sequencing was run under MinKNOW software (v24.11.8) (Oxford Nanopore Technologies). Nucleotide sequences were filtered with a minimum read length of ≥500 nt and a read quality of ≥7 for the nucleotide sequence filter and validation. Qualified reads in “Fast5” format were converted to “Fastq” format using the Nanopore Guppy basecaller (v 3.4.4). Sequences were assembled using a map‐to‐reference approach in Qiagen CLC Genomics Workbench (v20.0.4) (QIAGEN, CA, USA) with IAV reference sequences.

### 2.5. Phylogenetic and Genetic Analysis of swIAV

Maximum‐likelihood phylogenetic trees were constructed for the *HA*, *NA*, and internal genes of the Thai swIAVs. The phylogenetic analysis included more than 100 reference nucleotide sequences of swine, human, and avian influenza viruses retrieved from the GenBank and GISAID databases. The reference sequences were selected to represent the major lineages and clusters relevant to swIAV evolution. The maximum likelihood trees were generated using IQ‐TREE with 1000 bootstrap replicates. The numbers of *H1*, *H3*, *N1*, and *N2* gene sequences for phylogenetic analysis were 148, 142, 146, and 132, respectively. Trimmed sequences of *H1*, *H3*, *N1*, and *N2* genes were 1701, 1701, 1413, and 1410 base pairs, respectively. ModelFinder was used to identify the best‐fitting nucleotide substitution model. The best‐fit models of *H1*, *H3*, *N1*, and *N2* genes were GTR + F+I+G4, TVM + F+I+G4, GTR + F+I+G4, and GTR + F+I+G4, respectively. The trees were visualized using iTOL (v7.1) in rectangular mode, with distinct lineages designated by representative colors (avian lineage: red; Eurasian swine lineage: green; seasonal H3N2 or HU lineage: blue; CS lineage: pink).

The genetic constellation of each swIAV was determined by comparing each of the eight gene segments with reference sequences from the phylogenetic analysis, which included the pdmH1N1‐2009 lineage (pdmH1N1‐2009), the CS lineage, the Eurasia Avian‐like lineage (EA), the HU lineage, and the Triple Reassort swine lineage (TR). The analysis of genetic constellations was used to identify reassortment events and to assess the overall genetic diversity of circulating viruses. For genetic analysis, nucleotide and deduced amino acid sequence identities of all eight genes were compared with reference sequences from GenBank and GISAID. Sequence alignment was performed using MEGA12 to analyze genetic mutations and key amino acid residues in important genes (*HA, NA, PB2*, *M*, and *NS* genes).

### 2.6. Statistical Analysis

The association between swIAV prevalence across categorical variables, including pig age groups, provinces, and seasons, was assessed using the chi‐square test, with *p*  < 0.05 considered statistically significant. All statistical analyses were performed using GraphPad Prism (v9.0).

## 3. Results

### 3.1. Multiyear Swine IAV Surveillance in Thailand

**Figure 1:**
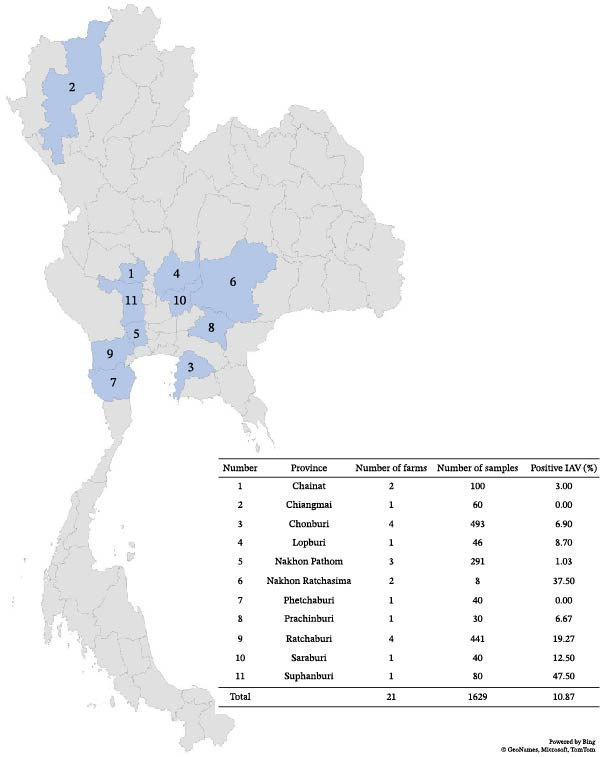
Map of Thailand, location of provinces, and number of pig farms for sample collection.

From 2019–2025, multiyear swIAV surveillance was conducted at 21 pig farms across 11 provinces in Thailand (Figure 1). A total of 1629 nasal swab samples were collected and screened for swIAV using real‐time RT‐PCR targeting the *M* gene. Out of the 1629 samples, 177 (10.87%) were identified as swIAV‐positive and 49 (3.01%) were identified as suspect (Table 1). The percentages of swIAV‐positive animals across individual farms ranged from 0% to 100%. Notably, farms with pigs exhibiting respiratory signs had a high swIAV positivity rate (95%–100%). The statistical analysis revealed an association between positivity rate and pig age (*χ* ^2^ = 50.40, *p*  < 0.05), with the highest prevalence observed in nursery pigs at 15.37% (138/898). Lower rates were seen in suckling pigs (≤4 weeks) at 9.94% (16/161), fattening pigs (>8–20 weeks) at 4.63% (17/367), and breeder pigs at 2.96% (6/203). Moreover, seasonality is statistically significant for the swIAV positivity rate (*χ* ^2^ = 14.53, *p*  < 0.05). Pigs tested during the rainy season (14.16%) have a higher chance of testing positive for swIAVs compared to those tested during the winter season (6.84%), with statistical significance (*p*  < 0.05). swIAV positivity by province ranged from 0% to 47.5%. The highest positive swIAV rates were observed in Suphanburi province (47.50%) and Ratchaburi province (19.27%) with statistical significance (*χ* ^2^ = 179.91, *p*  < 0.05; Table 2).

**Table 1:** Sample collection and swIAV detection in this study.

Year	# of sample collected	swIAV detection	
		Positive (suspected)/tested	Positive (%)
2019	123	20 (8)/123	16.26
2020	80	7 (1)/80	8.75
2021	8	3 (1)/8	37.5
2022	542	11 (7)/542	2.03
2023	526	120 (21)/526	22.81
2024	200	6 (8)/200	3.00
2025	150	10 (3)/150	6.67
Total	1629	177 (49)/1629	10.87

**Table 2:** swIAV detection in pig farms by province, age group, and season.

By provinces				
Provinces	# Farms	# Sample	# Positive	%
Chainat	2	100	3	3.00
Chiangmai	1	60	0	0
Chonburi	4	493	34	6.90
Lopburi	1	46	4	8.70
Nakhon Pathom	3	291	3	1.03
Nakhon Ratchasima	2	8	3	37.50
Phetchaburi	1	40	0	0
Prachinburi	1	30	2	6.67
Ratchaburi	4	441	85	19.27
Saraburi	1	40	5	12.50
Suphanburi	1	80	38	47.50
Total	21	1629	177	10.87
Chi‐square statistic (*χ* ^2^) = 179.91, *df* = 10 (*p*‐value < 0.05)				

By age group				
Age group	Age (weeks)	# Sample	# Positive	%
Suckling pigs	≤4 weeks	161	16	9.94
Nursery pigs	>4–8 weeks	898	138	15.37
Fattening pigs	>8–20 weeks	367	17	4.63
Breeder pigs	Adult	203	6	2.96
Chi‐square statistic (*χ* ^2^) = 50.40, *df* = 3 (*p*‐value < 0.05)				

By Season				
Season	Duration	#Samples	#Positive	*p*‐Value ^∗^(%)
Winter	November–January	395	27	6.84
Summer	February–May	556	54	9.71
Rainy	June–October	678	96	14.16
Chi‐square statistic (*χ* ^2^) = 14.53, *df* = 2 (*p*‐value < 0.05)				

^∗^Indicates statistical significance.

### 3.2. Whole Genome Characterization and Genetic Constellation of Thai‐SwIAVs

The swIAV‐positive and suspected samples were subjected to virus isolation through egg inoculation and/or cell culture. In brief, the positive and suspect samples from real‐time RT‐PCR screening (*n* = 226) were subjected to egg inoculation. Twenty‐two samples were successfully isolated by egg inoculation (9.73% isolation rate, 22/226). Then, the egg‐inoculation (two passages) negative samples (*n* = 204) were subjected to MDCK cell culture. Eight samples were successfully isolated by MDCK cell culture (3.92% isolation rate, 8/204). In total, 30 SIVs were isolated (isolation rate: 13.27%, 30/226). Whole‐genome sequencing was performed on all isolated samples (*n* = 30) (Supporting Information 2: Table S1). The whole‐genome sequences of 30 Thai‐swIAVs were deposited in GenBank and FluDB under accession numbers PX444173–PX444412 (Table 3). Subtyping of swIAVs revealed three circulating subtypes among the 30 isolates: swIAV‐H1N1 (*n* = 14) and swIAV‐H3N2 (*n* = 13) were detected during 2019–2025, while swIAV‐H1N2 (*n* = 3) was detected only in 5–6‐week‐old pigs on a single farm in 2023. Notably, swIAV‐H1N2 in Thailand had not been reported in the FluDB and GISAID databases since 2013.

**Table 3:** Description of Thai‐swIAV characterized in this study.

Virus	Collection date	Age	Province	Subtype	GenBank Accession umber
A/swine/Thailand/CU24248/2019	Apr‐19	4 weeks‐old	Ratchaburi	H1N1	PX444173–180
A/swine/Thailand/CU24758/2019	Aug‐19	8 weeks‐old	Ratchaburi	H1N1	PX444213–220
A/swine/Thailand/CU24760/2019	Aug‐19	8 weeks‐old	Ratchaburi	H1N1	PX444221–228
A/swine/Thailand/CU26137/2020	Dec‐20	4 weeks‐old	Ratchaburi	H1N1	PX444261–268
A/swine/Thailand/CU30318/2022	Oct‐22	4 weeks‐old	Ratchaburi	H1N1	PX444285–292
A/swine/Thailand/CU30334/2022	Oct‐22	Gilt	Ratchaburi	H1N1	PX444293–300
A/swine/Thailand/CU30335/2022	Oct‐22	Gilt	Ratchaburi	H1N1	PX444301–308
A/swine/Thailand/CU30359/2022	Oct‐22	Gilt	Ratchaburi	H1N1	PX444309–316
A/swine/Thailand/CU32449/2023	Apr‐23	5 weeks‐old	Ratchaburi	H1N1	PX444333–340
A/swine/Thailand/CU32453/2023	Apr‐23	5 weeks‐old	Ratchaburi	H1N1	PX444341–348
A/swine/Thailand/CU33613/2023	Sep‐23	4 weeks‐old	Suphanburi	H1N1	PX444373–380
A/swine/Thailand/CU33622/2023	Sep‐23	4 weeks‐old	Suphanburi	H1N1	PX444381–388
A/swine/Thailand/CU33637/2023	Sep‐23	5 weeks‐old	Suphanburi	H1N1	PX444389–396
A/swine/Thailand/CU35124/2024	Feb‐24	8 weeks‐old	Chonburi	H1N1	PX444397–404
A/swine/Thailand/CU33552/2023	Sep‐23	5 weeks‐old	Ratchaburi	H1N2	PX444349–356
A/swine/Thailand/CU33558/2023	Sep‐23	5 weeks‐old	Ratchaburi	H1N2	PX444357–364
A/swine/Thailand/CU33569/2023	Sep‐23	6 weeks‐old	Ratchaburi	H1N2	PX444365–372
A/swine/Thailand/CU24251/2019	Apr‐19	4 weeks‐old	Ratchaburi	H3N2	PX444181–188
A/swine/Thailand/CU24271/2019	Apr‐19	8 weeks‐old	Ratchaburi	H3N2	PX444189–196
A/swine/Thailand/CU24272/2019	Apr‐19	8 weeks‐old	Ratchaburi	H3N2	PX444197–204
A/swine/Thailand/CU24274/2019	Apr‐19	8 weeks‐old	Ratchaburi	H3N2	PX444205–212
A/swine/Thailand/CU24926/2019	Nov‐19	6 weeks‐old	Ratchaburi	H3N2	PX444229–236
A/swine/Thailand/CU24929/2019	Nov‐19	6 weeks‐old	Ratchaburi	H3N2	PX444237–244
A/swine/Thailand/CU25074/2020	Feb‐20	6 weeks‐old	Ratchaburi	H3N2	PX444245–252
A/swine/Thailand/CU26133/2020	Dec‐20	4 weeks‐old	Ratchaburi	H3N2	PX444253–260
A/swine/Thailand/CU28095/2021	Nov‐21	10 weeks‐old	Nakhon Ratchasima	H3N2	PX444269–276
A/swine/Thailand/CU28100/2021	Nov‐21	10 weeks‐old	Nakhon Ratchasima	H3N2	PX444277–284
A/swine/Thailand/CU31947/2023	Mar‐23	2 weeks‐old	Lopburi	H3N2	PX444317–324
A/swine/Thailand/CU31994/2023	Mar‐23	Breeder	Saraburi	H3N2	PX444325–332
A/swine/Thailand/CU36481/2025	Mar‐25	6 weeks‐old	Chonburi	H3N2	PX444405–412

The genetic constellation of the Thai‐swIAVs was identified through phylogenetic analysis of the eight gene segments, revealing complex genetic reassortment patterns that resulted in multiple distinct genotypes (Tables 4–6). Our results showed that four genotypes of the Thai‐swIAV‐H1N1 were identified. There were three reassortant swIAV‐H1N1 (rH1N1), including rH1N1 (7pdm + 1CS) (*n* = 4), rH1N1 (5pdm + 2EA + 1CS) (*n* = 4), rH1N1 (6pdm + 1EA + 1CS) (*n* = 5), and endemic swIAV‐H1N1 (enH1N1; 8EA; *n* = 1). In detail, the rH1N1 (7pdm + 1CS) genotype, isolated in 2019–2020, comprised the H1 gene from the CS lineage and seven genes (*N1*, *PB2*, *PB1*, *PA, NP, M*, and *NS*) from the pdmH1N‐2009 backbone (Table 4). Genotype rH1N1 (6pdm + 1EA + 1CS), isolated in 2023, consisted of the H1 gene from CS, the *N1* gene from EA, and the other six internal genes (*PB2*, *PB1*, *PA, NP, M*, and *NS* genes) from the pdmH1N1‐2009 backbone. The rH1N1 (5pdm + 2EA + 1CS) genotype, recovered in 2022, comprised the H1 gene from CS, the *N1* and *M* genes from EA, and the other five internal genes (PB2, PB1, PA, NP, and NS) from pdmH1N1‐2009. It is noted that this genetic constellation pattern (5pdm + 2EA + 1CS) has never been reported in Thailand. Genotype enH1N1 (8EA) was isolated in 2024; all genes were acquired from EA and were closely related to those of swIAVs isolated in Denmark in 2020 and 2021. This enH1N1 (8EA) genotype has never been reported in the Thai pig population (Table 4).

**Table 4:** Genetic constellation of Thai‐swIAV‐H1N1 characterized in this study.

Virus	Location	Year	Genotype	Genetic constellation								
Reference viruses				PB2	PB1	PA	HA	NP	NA	M	NS	Ref
A/California/04/09	Ref	2009	pdmH1N1‐2009	pdm	pdm	pdm	pdm	pdm	pdm	pdm	pdm	^a^
A/swine/Shandong/1207/2016	Ref	2016	G4	pdm	pdm	pdm	EA	pdm	EA	pdm	TR	^b^
A/swine/Ratchaburi/NIAH1481/2000	Thailand	2000	eH1N1 (7EA + 1CS)	EA	EA	EA	CS	EA	EA	EA	EA	^c^
A/swine/Chachoengsao/NIAH587/2005	Thailand	2005	eH1N1 (6EA + 2CS)	EA	EA	EA	CS	EA	EA	EA	CS	^c^
A/sw/Thailand/CU‐RA20/2009	Thailand	2009	eH1N1 (6EA + 2CS)	EA	EA	EA	CS	EA	EA	EA	CS	^d^
A/swine/Saraburi/NIAH100761–22/2009	Thailand	2009	eH1N1 (7EA + 1CS)	EA	EA	EA	CS	EA	EA	EA	EA	^c^
A/swine/Thailand/CU‐PS73/2010	Thailand	2010	eH1N1 (6EA + 2CS)	EA	EA	EA	CS	EA	EA	EA	CS	^e, f^
A/swine/Thailand/CU_SA43/2010	Thailand	2010	rH1N1 (7pdm + 1EA)	pdm	pdm	pdm	pdm	pdm	EA	pdm	pdm	^g^
A/swine/Chachoengsao/NIAH105583–052–92/2012	Thailand	2012	rH1N1 (6pdm + 2EA)	pdm	pdm	pdm	EA	pdm	EA	pdm	pdm	^h^
A/swine/Thailand/CU_S3795N/2013	Thailand	2013	rH1N1 (6pdm + 1EA + 1CS)	pdm	pdm	pdm	CS	pdm	EA	pdm	pdm	^i^
A/swine/Thailand/CU3770/2017	Thailand	2017	rH1N1 (6pdm + 1EA + 1CS)	pdm	pdm	pdm	CS	pdm	EA	pdm	pdm	^j^
A/swine/Thailand/CU21299/2018	Thailand	2018	rH1N1 (7pdm + 1CS)	pdm	pdm	pdm	CS	pdm	pdm	pdm	pdm	^j^
Viruses in this study												
A/swine/Thailand/CU24248/2019	Ratchaburi	2019	rH1N1 (7pdm + 1CS)	pdm	pdm	pdm	CS	pdm	pdm	pdm	pdm	—
A/swine/Thailand/CU24758/2019	Ratchaburi	2019	rH1N1 (7pdm + 1CS)	pdm	pdm	pdm	CS	pdm	pdm	pdm	pdm	—
A/swine/Thailand/CU24760/2019	Ratchaburi	2019	rH1N1 (7pdm + 1CS)	pdm	pdm	pdm	CS	pdm	pdm	pdm	pdm	—
A/swine/Thailand/CU26137/2020	Ratchaburi	2020	rH1N1 (7pdm + 1CS)	pdm	pdm	pdm	CS	pdm	pdm	pdm	pdm	—
A/swine/Thailand/CU30318/2022	Ratchaburi	2022	rH1N1 (5pdm + 2EA + 1CS)	pdm	pdm	pdm	CS	pdm	EA	EA	pdm	—
A/swine/Thailand/CU30334/2022	Ratchaburi	2022	rH1N1 (5pdm + 2EA + 1CS)	pdm	pdm	pdm	CS	pdm	EA	EA	pdm	—
A/swine/Thailand/CU30335/2022	Ratchaburi	2022	rH1N1 (5pdm + 2EA + 1CS)	pdm	pdm	pdm	CS	pdm	EA	EA	pdm	—
A/swine/Thailand/CU30359/2022	Ratchaburi	2022	rH1N1 (5pdm + 2EA + 1CS)	pdm	pdm	pdm	CS	pdm	EA	EA	pdm	—
A/swine/Thailand/CU32449/2023	Ratchaburi	2023	rH1N1 (6pdm + 1EA + 1CS)	pdm	pdm	pdm	CS	pdm	EA	pdm	pdm	—
A/swine/Thailand/CU32453/2023	Suphanburi	2023	rH1N1 (6pdm + 1EA + 1CS)	pdm	pdm	pdm	CS	pdm	EA	pdm	pdm	—
A/swine/Thailand/CU33613/2023	Suphanburi	2023	rH1N1 (6pdm + 1EA + 1CS)	pdm	pdm	pdm	CS	pdm	EA	pdm	pdm	—
A/swine/Thailand/CU33622/2023	Suphanburi	2023	rH1N1 (6pdm + 1EA + 1CS)	pdm	pdm	pdm	CS	pdm	EA	pdm	pdm	—
A/swine/Thailand/CU33637/2023	Ratchaburi	2023	rH1N1 (6pdm + 1EA + 1CS)	pdm	pdm	pdm	CS	pdm	EA	pdm	pdm	—
A/swine/Thailand/CU35124/2024	Chonburi	2024	enH1N1 (8EA)	EA	EA	EA	EA	EA	EA	EA	EA	—

Abbreviations: CS, Classical swine lineage/North American swine lineage; EA, Eurasia avian‐like lineage/Eurasian swine lineage; HU, human seasonal lineage/Human‐like lineage; HUa, human‐like A sublineage; HUb, human‐like B sublineage; G4, genotype of IAV contains genes from pdm (*PB2*, *PB1*, *PA, NP*, and *M* genes), triple reassortant (*NS* gene), and Eurasian avian‐like swine (*HA* and *NA* genes); pdm, Pandemic‐H1N1‐2009 lineage; TR, triple reassort swine lineage.

^a^[9].

^b^[28].

^c^[33].

^d^[34].

^e^[35].

^f^[36].

^g^[22].

^h^GISAID.

i[19].

^j^[37].

**Table 5:** Genetic constellation of Thai‐swIAV‐H1N2 characterized in this study.

Virus	Location	Year	Genotype	Genetic constellation								
Reference viruses				PB2	PB1	PA	HA	NP	NA	M	NS	Ref
A/swine/Saraburi/NIAH13021/2005	Thailand	2005	H1N2	EA	EA	EA	CS	EA	EA	EA	CS	^b^
A/swine/Thailand/CU‐CHK4/2009	Thailand	2009	H1N2	—	—	—	CS	EA	—	—	—	^c^
A/swine/Thailand/CU‐Cbo/2009	Thailand	2009	H1N2	EA	EA	—	CS	EA	—	EA	CS	^c^
A/swine/Thailand/CU‐CT43/2011	Thailand	2011	rH1N2	—	pdm	—	pdm	pdm	HU	pdm	pdm	^a^
A/swine/Thailand/CU‐CT63/2011	Thailand	2011	rH1N2	pdm	pdm	—	pdm	pdm	HU	pdm	pdm	^a^
A/swine/Thailand/CU‐CT83/2011	Thailand	2011	rH1N2	pdm	pdm	—	pdm	pdm	HU	pdm	pdm	^a^
A/swine/Thailand/CU‐S3073N/2011	Thailand	2011	rH1N2	—	pdm	pdm	pdm	pdm	HUb	pdm	pdm	^c^
A/swine/Thailand/CU‐S3631N/2012	Thailand	2012	rH1N2	pdm	—	pdm	CS	pdm	—	—	—	^c^
A/swine/Thailand/NIAH105583–052–96/2012	Thailand	2012	rH1N2 (6pdm + 1EA + 1HU)	pdm	pdm	pdm	EA	pdm	HUa	pdm	pdm	^c^
Viruses in this study												
A/swine/Thailand/CU33552/2023	Ratchaburi	2023	rH1N2 (6pdm + 1EA + 1HU)	pdm	pdm	pdm	EA	pdm	HUa	pdm	pdm	—
A/swine/Thailand/CU33558/2023	Ratchaburi	2023	rH1N2 (6pdm + 1EA + 1HU)	pdm	pdm	pdm	EA	pdm	HUa	pdm	pdm	—
A/swine/Thailand/CU33569/2023	Ratchaburi	2023	rH1N2 (6pdm + 1EA + 1HU)	pdm	pdm	pdm	EA	pdm	HUa	pdm	pdm	—

Abbreviations: CS, classical swine lineage/North American swine lineage; EA, Eurasia avian‐like lineage/Eurasian swine lineage; HU, human seasonal lineage/human‐like lineage; HUa, human‐like A sublineage; HUb, human‐like B sublineage; pdm, Pandemic‐H1N1‐−2009 lineage.

^a^[35].

^b^[36].

^c^GISAID.

**Table 6:** Genetic constellation of Thai‐swIAV‐H3N2 characterized in this study.

Virus	Location	Year	Genotype	Genetic constellation								
Reference viruses				PB2	PB1	PA	HA	NP	NA	M	NS	Ref
A/swine/Chachoengsao/2003	Thailand	2003	eH3N2 (6EA + 2CS)	EA	EA	EA	EA	CS	EA	EA	CS	^a^, ^b^
A/swine/Udon Thani/NIAH464/2004	Thailand	2004	eH3N2 (8EA)	EA	EA	EA	EA	EA	EA	EA	EA	^a, b^
A/swine/Ratchaburi/NIAH59/2004	Thailand	2004	eH3N2 (5EA + 2HU + 1CS)	EA	EA	EA	HUa	EA	HUa	EA	CS	^a, b^
A/swine/Thailand/KU5.1/2004	Thailand	2004	eH3N2 (4EA + 2HU + 2CS)	EA	EA	EA	HUb	CS	HUb	EA	CS	^f, e^
A/swine/Saraburi/NIAH107725–28/2008	Thailand	2008	eH3N2 (4EA + 2HU + 2CS)	EA	EA	EA	HUb	CS	HUb	EA	CS	^a, b^
A/swine/N.Pathom/NIAH586–1/2005	Thailand	2005	eH3N2 (7EA + 1CS)	EA	EA	EA	EA	CS	EA	EA	EA	^a, b^
A/swine/Chonburi/NIAH106952–026/2011	Thailand	2011	rH3N2 (6pdm + 2HU)	pdm	pdm	pdm	HUa	pdm	HUa	pdm	pdm	^a, f^
A/swine/Thailand/CU‐S3689N/2013	Thailand	2013	rH3N2 (6pdm + 2HU)	pdm	pdm	pdm	HUb	pdm	HUb	pdm	pdm	^c^
A/swine/Thailand/CU3790/2017	Thailand	2017	rH3N2 (6pdm + 2HU)	pdm	pdm	pdm	HUb	pdm	HUb	pdm	pdm	^d^
A/swine/Thailand/CU20226/2017	Thailand	2017	rH3N2 (6pdm + 2HU)	pdm	pdm	pdm	HUb	pdm	HUb	pdm	pdm	^d^
A/swine/Thailand/CU22337/2018	Thailand	2018	rH3N2 (6pdm + 2HU)	pdm	pdm	pdm	HUa	pdm	HUa	pdm	pdm	^d^
Viruses in this study												
A/swine/Thailand/CU24251/2019	Ratchaburi	2019	rH3N2 (6pdm + 2HU)	pdm	pdm	pdm	HUa	pdm	HUa	pdm	pdm	—
A/swine/Thailand/CU24271/2019	Ratchaburi	2019	rH3N2 (6pdm + 2HU)	pdm	pdm	pdm	HUa	pdm	HUa	pdm	pdm	—
A/swine/Thailand/CU24272/2019	Ratchaburi	2019	rH3N2 (6pdm + 2HU)	pdm	pdm	pdm	HUa	pdm	HUa	pdm	pdm	—
A/swine/Thailand/CU24274/2019	Ratchaburi	2019	rH3N2 (6pdm + 2HU)	pdm	pdm	pdm	HUa	pdm	HUa	pdm	pdm	—
A/swine/Thailand/CU24926/2019	Ratchaburi	2019	rH3N2 (4pdm + 2CS + 2HU)	pdm	pdm	pdm	HUb	CS	HUb	pdm	CS	—
A/swine/Thailand/CU24929/2019	Ratchaburi	2019	rH3N2 (4pdm + 2CS + 2HU)	pdm	pdm	pdm	HUb	CS	HUb	pdm	CS	—
A/swine/Thailand/CU25074/2020	Ratchaburi	2020	rH3N2 (6pdm + 2HU)	pdm	pdm	pdm	HUa	pdm	HUa	pdm	pdm	—
A/swine/Thailand/CU26133/2020	Ratchaburi	2020	rH3N2 (6pdm + 2HU)	pdm	pdm	pdm	HUa	pdm	HUa	pdm	pdm	—
A/swine/Thailand/CU28095/2021	N. Ratchasima	2021	eH3N2 (6EA + 2HU)	EA	EA	EA	HU	EA	HU	EA	EA	—
A/swine/Thailand/CU28100/2021	N. Ratchasima	2021	eH3N2 (6EA + 2HU)	EA	EA	EA	HU	EA	HU	EA	EA	—
A/swine/Thailand/CU31947/2023	Lopburi	2023	rH3N2 (6pdm + 2HU)	pdm	pdm	pdm	HUa	pdm	HUa	pdm	pdm	—
A/swine/Thailand/CU31994/2023	Saraburi	2023	rH3N2 (6pdm + 2HU)	pdm	pdm	pdm	HUa	pdm	HUa	pdm	pdm	—
A/swine/Thailand/CU36481/2025	Chonburi	2025	rH3N2 (6pdm + 2HU)	pdm	pdm	pdm	HUa	pdm	HUa	pdm	pdm	—

Abbreviations: CS, classical swine lineage/North American swine lineage; EA, Eurasia avian‐like lineage/Eurasian swine lineage; HU, human seasonal lineage/human‐like lineage; HUa, human‐like A sublineage; HUb, human‐like B sublineage; pdm, Pandemic‐H1N1‐−2009 lineage.

^a^[33].

^b^[36].

^c^[19].

^d^[38].

^e^[18].

^f^[39].

The genetic constellation of Thai‐swIAV‐H1N2 showed the reassortant swIAV‐H1N2 (rH1N2) genotype (6pdm + 1EA + 1HU) (*n* = 3), which was recovered in 2023. This genotype consisted of H1 from EA, N2 from the human seasonal H3N2 lineage, sublineage human‐like A (HUa), and the internal six genes from the pdmH1N1‐2009 backbone. This genotype has previously been reported as the most recent Thai swIAV‐H1N2 sequence submitted to the GISAID database (EpiFlu) (Table 5). The genetic constellation of Thai‐swIAV‐H3N2 identified two reassortant swIAV‐H3N2 (rH3N2): (6pdm + 2HU) (*n* = 9), (4pdm + 2CS + 2HU; *n* = 2), and endemic swIAV‐H3N2 (enH3N2) (6EA + 2HU) (*n* = 2). In detail, the rH3N2 (6pdm + 2HU) genotype, obtained in 2019, 2020, 2023, and 2025, comprised the H3 and N2 genes from the HU lineage, sublineage HUa, and six internal genes from the pdmH1N1‐2009 backbone. rH3N2 (4pdm + 2CS + 2HU), isolated in 2019, consists of the *H3* and *N2* genes from the HU lineage, sublineage human‐like B (HUb), the *NP* and *NS* genes from the CS lineage, and the *PB2*, *PB1*, *PA*, and *M* genes from the pdmH1N1‐2009 lineages. Notably, this genotype, rH3N2 (4pdm + 2CS + 2HU) is a novel genotype in Thailand and has never been reported in the GenBank Fludb and GISAID databases [18, 19, 33, 35, 38, 39]. Although enH3N2 (6EA + 2HU) was identified in this study in 2021, our findings indicated that enH3N2 continued to circulate on pig farms (Table 6).

### 3.3. Phylogenetic Analysis of Thai‐SwIAVs

**Figure 2:**
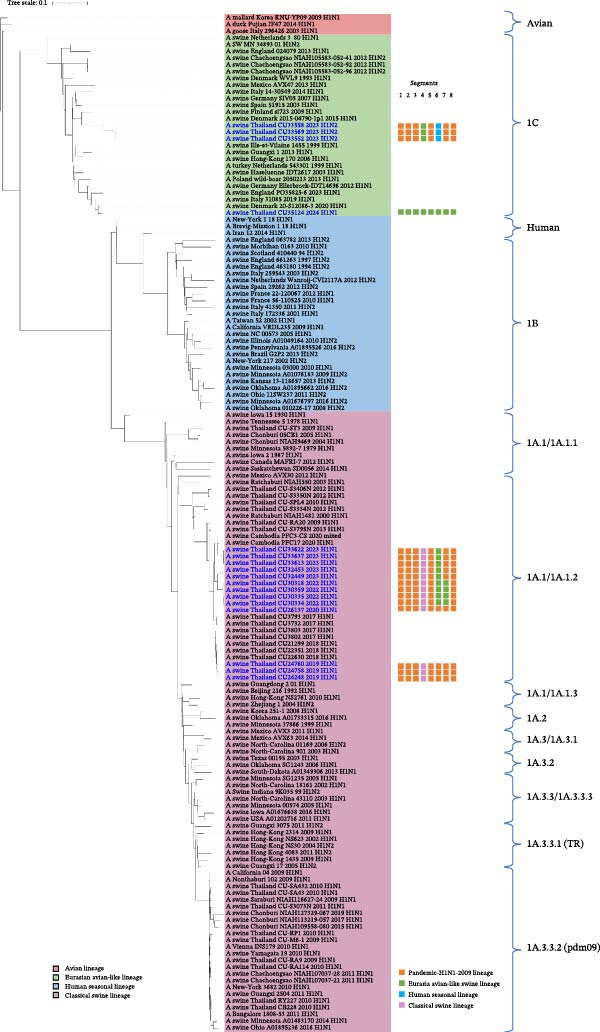
The maximum likelihood tree of the *H1* gene. Color represents the lineage of the *H1* gene, including red (Avian lineage), green (Eurasian avian‐like lineage; EA), blue (human seasonal lineage; HU), and pink (classical swine lineage; CS). Virus names in blue indicate Thai‐swIAV‐H1N1 in the study.

**Figure 3:**
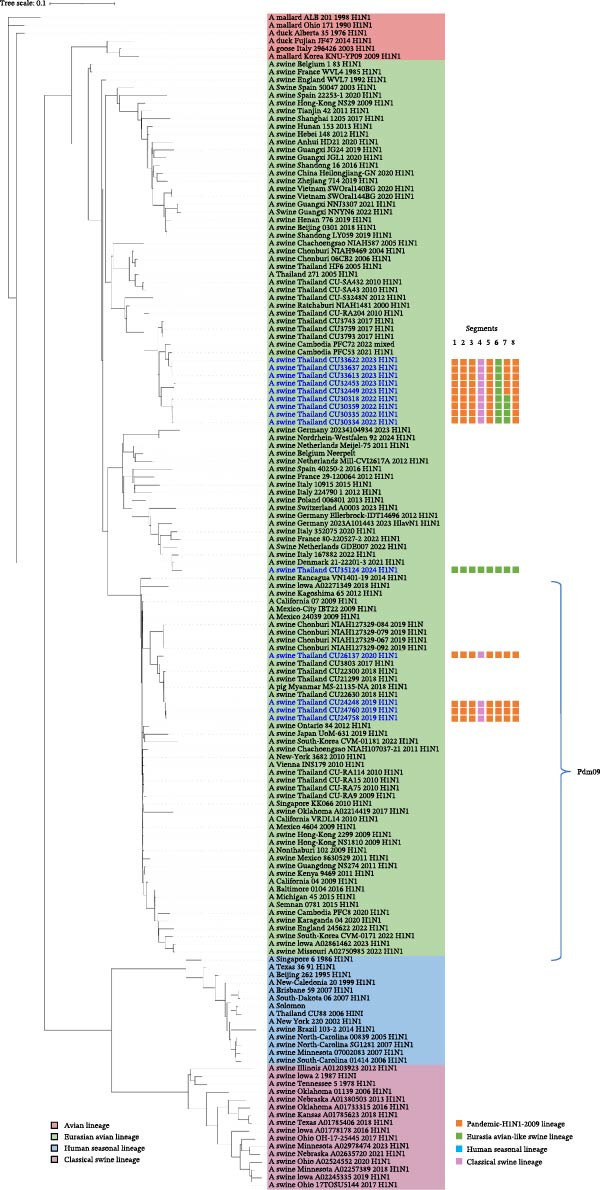
The maximum likelihood tree of the *N1* gene. Color represents the lineage of the *N1* gene, including red (Avian lineage), green (Eurasian avian‐like lineage; EA), blue (human seasonal lineage (HU), and pink (classical swine lineage; CS). Virus names in blue indicate Thai‐swIAV‐H1N1 in the study.

**Figure 4:**
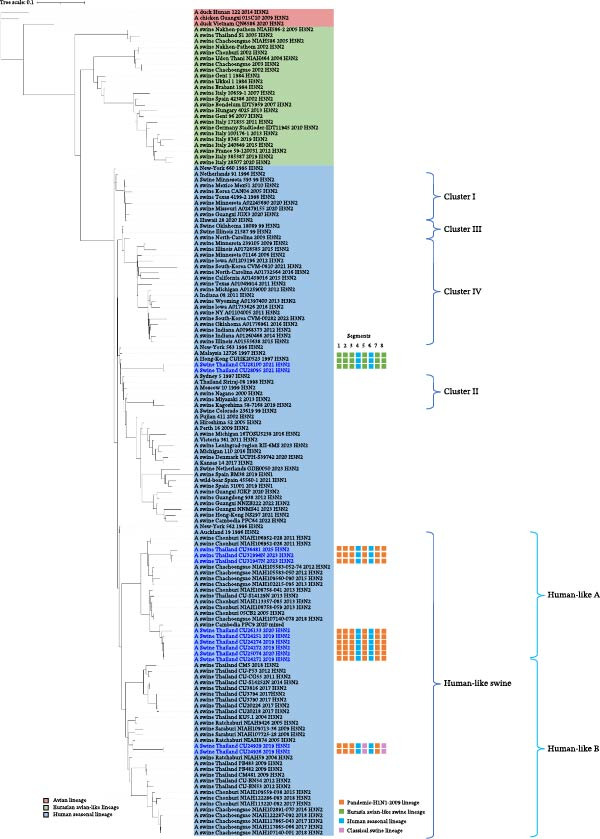
The maximum likelihood tree of the *H3* gene. Color represents the lineage of the *H3* gene, including red (Avian lineage), green (Eurasian avian‐like lineage; EA), and blue (human seasonal lineage; HU). Virus names in blue indicate Thai‐swIAV‐H3N2 in the study.

**Figure 5:**
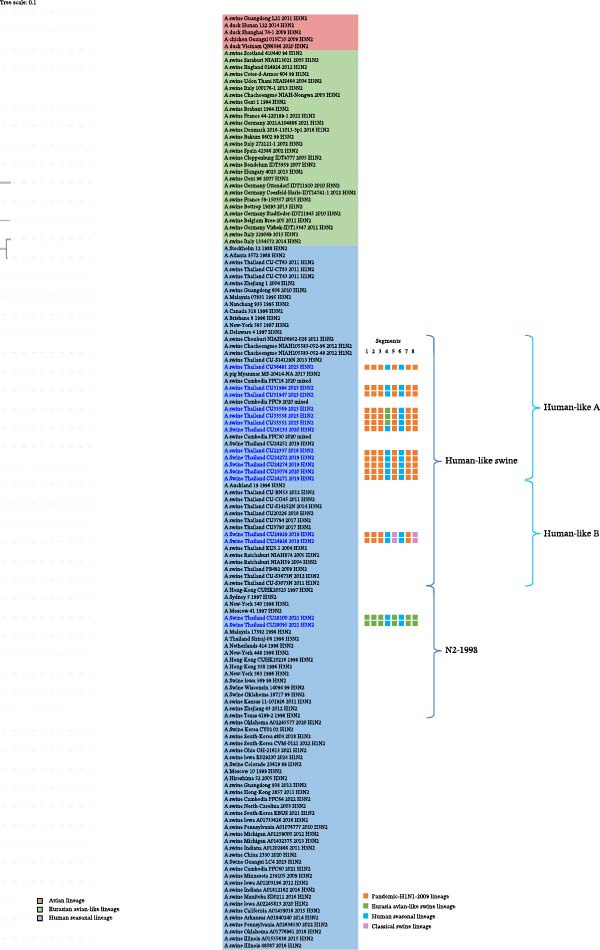
The maximum likelihood tree of the *N2* gene. Color represents the lineage of the *N2* gene, including red (Avian lineage), green (Eurasian avian‐like lineage; EA), and blue (human seasonal H3N2 lineage; HU). Virus names in blue indicate Thai‐swIAV‐H1N2 and swIAV‐H3N2 in the study.

The genetic relationships among the Thai‐swIAVs are shown in Figures 2–5 and Supporting Information 1: Figures S2–S7. Phylogenetic analysis of the *HA* and *NA* genes provided detailed lineage information. For instance, phylogenetic analysis of H1 showed that 13 Thai‐swIAV‐H1N1 clustered within the CS, alpha sublineage (1A.1.2), consistent with previous reports of Thai‐swIAVs and Cambodia‐swIAVs during 2009–2020. In contrast, the H1 of Thai‐swIAV‐H1N2 (*n* = 3) (CU33552, CU33558, and CU33569) and one Thai‐swIAV‐H1N1 (CU35124) grouped within the EA, sublineage 1C (Figure 2). Phylogenetic analysis of N1 showed that nine rH1N1 and one enH1N1 clustered in the EA. Four rH1N1 were grouped into pdmH1N1‐2009, similar to the previous Thai swIAVs (Figure 3). For phylogenetic analysis of H3, Thai‐swIAV‐H3N2 (*n* = 13) clustered within HU but across different sublineages A and B (HUa and HUb). In detail, nine rH3N2 were clustered within HUa, and two rH3N2 were clustered into HUb. Additionally, two enH3N2 (CU28095 and CU28100) were grouped into the seasonal H3N2 lineage reported in 1996–1998 (HU; Figure 4). In the phylogenetic analysis of N2, nine rH3N2 grouped with HUa and two H3N2 (CU24926 and CU24929) clustered into HUb. While two enH3N2 (CU28095 and CU28100) clustered into the seasonal H3N2 lineage (N2—1998 sublineage), an older sublineage of the HU lineage (Figure 5). Note that phylogenetic trees for the six internal genes (*PB2*, *PB1*, *PA, NP, M*, and *NS*) were provided and used to analyze the genetic constellations of the viruses (Supporting Information 1: Figures S2–S7).

### 3.4. Genetic Analysis of Thai‐SwIAVs

Analysis of key amino acid substitutions at the antigenic site, receptor‐binding site (RBS), and HA cleavage site of the H1 and H3 of Thai‐swIAVs is shown in Tables 7 and 8. Genetic analysis of the *H1* gene showed that the HA cleavage site of the Thai swIAV‐H1N1 contains PSIQSRGLF, identical to that of the pdmH1N1−2009 and previous Thai‐swIAVs (2003–2018). The HA RBS showed D190 and D225, indicating the retention of receptor‐binding properties for the avian receptor (α2,3‐linked sialic acid receptors) [40, 41]. However, two swIAV‐H1N1 (CU24248 and CU24758) exhibited D190 and 225G, suggesting preferential binding to both avian and mammalian receptors (α2,6‐linked sialic acid receptors) (Table 7). All the Thai swIAV‐H3N2 showed 226I/V and 228 S, suggesting preferential binding to the mammalian receptor [42]. Analysis of antigenic sites showed that some swIAV‐H1N1 and swIAV‐H3N2 exhibited slightly different amino acid patterns in the antigenic sites (Sa, Sb, Ca1, Cb, A, C, D, and E) compared to previously reported Thai‐swIAVs, indicating antigenic drift. It is noted that amino acid substitutions in those antigenic sites caused antigenic drift in human influenza evolution [43, 44].

**Table 7:** Genetic analysis of the *H1* gene of Thai‐swIAVs in this study.

Viruses	HA cluster	Amino acid sequence alignment of *H1* gene													
		Antigenic site										Receptor binding site		HA cleavage site	
		Sa			Sb	Ca1			Ca2		Cb				
		128–129 ^∗^	156–160 ^∗^	162–167 ^∗^	187–198 ^∗^	169–173 ^∗^	206–208 ^∗^	238–240 ^∗^	140–145 ^∗^	224–225 ^∗^	78–83 ^∗^	190 ^∗^	225 ^∗^		325–333 ^∗^
A/California/04/09	1A.3.3.2	PN	KKGNS	PKLSKS	TSADQQSIYQNA	INDKG	GSS	EPG	PHAGAK	RD	LSTASS	D	D		PSIQSRGLF
A/swine/Thailand/CU‐S3073/11	1A.3.3.2	PN	KKENS	PKLSKS	TSADQQSIYQNA	INDKG	GSS	EPG	PHAGAK	RD	LSTASS	D	D		PSIQSRGLF
A/swine/Thailand/CU‐RA9/2009	1A.3.3.2	PN	KKGNS	PKLSKS	TSADQQSLYQNA	INDKG	GTS	EPG	PHAGAK	RD	LSTASS	D	D		PSIQSRGLF
A/swine/Chonburi/NIAH127329‐067/2019	1A.3.3.2	PN	KKGNS	PKLSKS	TSTDQQSLYQNA	INNKG	GSS	EPG	PRAGAR	RD	LSAVSS	D	D		PSIQSRGLF
A/swine/Ratchaburi/NIAH550/2003	1A.1	PN	KKGNS	PKLSKS	TNNDQQTIYQNN	VNNKG	GSS	EPG	PYAGAN	RD	LFTASS	D	D		PSIQSRGLF
A/swine/Chonburi/NIAH9469/04	1A.1.1	PN	KKGNS	PKLRKA	TNTDQQSLYQNA	VNNKK	GSS	EPG	PYAGTN	RG	LFAVNS	D	G		PSIQSRGLF
A/Swine/Thailand/CU‐S3334N/12	1A.1.2	PN	KKGNS	PKLSKS	TSTDQQSLYQNA	VNNKK	SSS	EPG	HHAGAK	RD	LFKANS	D	D		PSIQSRGLF
A/Swine/Thailand/CU‐S3350N/12	1A.1.2	PN	KKANS	PKLSKS	TITDQQSLYQNT	LNNKK	SSS	EPG	PHAGAN	RD	LFRANS	D	D		PSIQSRGLF
A/Swine/Thailand/CU‐S3629N/12	1A.1.2	PN	KKENS	PKISKS	TSNDQQSLYQNA	FNNKG	SSS	KPG	PYAGAN	RD	LFNANS	D	D		PSIQSRGLF
A/swine/Thailand/CU3803/2017	1A.1.2	PN	KKENS	PKLSKS	TSNDQQVLYQNA	FNNRG	SSS	KPE	PYAGAN	RD	LFNANS	D	D		PSIQSRGLF
A/swine/Thailand/CU22351/2018	1A.1.2	PN	KKENS	PKLSKS	TSNDQQVLYQNA	FNNRG	SSS	KPE	PYAGAN	RD	LFNANS	D	D		PSIQSRGLF
A/swine/Thailand/CU22630/2018	1A.1.2	PN	KKENS	PKLSKS	TSNDQQVLYQNA	FNNRG	SSS	KPE	PYAGAN	RG	LFNANS	D	G		PSIQSRGLF
A/swine/Denmark/2015/04790/1p1/2015	1C	PE	KKG‐S	PKISKS	NYSDQQTLYQNN	TNDKG	SSK	TPE	SHSGAP	RE	LLTANS	D	E		PSIQSRGLF
This Study															
A/Swine/Thailand/CU24248/2019	1A.1.2	PN	KKENS	PKLSKS	TSNDQQVLYQNA	FNNRG	SSS	KPE	PYAGAN	RG	LFNANS	D	G		PSIQSRGLF
A/Swine/Thailand/CU24758/2019	1A.1.2	PN	KKENS	PKLSKS	TSNDQQVLYQNA	FNNRG	SSS	KPE	PYAGAN	RG	LFNANS	D	G		PSIQSRGLF
A/Swine/Thailand/CU24760/2019	1A.1.2	PN	KKENS	PKLSKS	TSNDQQVLYQNA	FNNRG	SSS	KPE	PYAGAN	RD	LFNANS	D	D		PSIQSRGLF
A/Swine/Thailand/CU26137/2020	1A.1.2	PN	KKGNS	PKLSKS	TNNDQQVLYQNA	FNNKG	SSS	KPE	PHAGAN	RD	LLNANS	D	D		PSIQSRGLF
A/Swine/Thailand/CU30318/2022	1A.1.2	PN	KKGNS	PKLSKS	TSNDQQALYQNA	FNNKG	SSS	KPE	PYAGAN	RD	LFNASS	D	D		PSIQSRGLF
A/Swine/Thailand/CU30334/2022	1A.1.2	PN	KKGNS	PKLSKS	TSNDQQALYQNA	FNNKG	SSS	KPE	PYAGAN	RD	LFNANS	D	D		PSIQSRGLF
A/Swine/Thailand/CU30335/2022	1A.1.2	PN	KKGNS	PKLSKS	TSNDQQALYQNA	FNNKG	SSS	KPE	PYAGAN	RD	LFNANS	D	D		PSIQSRGLF
A/Swine/Thailand/CU30359/2022	1A.1.2	PN	KKGNS	PKLSKS	TSNDQQALYQNA	FNNKG	SSS	KPE	PYAGAN	RD	LFNANS	D	D		PSIQSRGLF
A/Swine/Thailand/CU32449/2023	1A.1.2	PN	KKENS	PKLSKS	TSNDQQALYQNA	FNNKG	SSS	KPE	PYAGAN	RD	LFNANS	D	D		PSIQSRGLF
A/Swine/Thailand/CU32453/2023	1A.1.2	PN	KKENS	PKLSKS	TSNDQQALYQNA	FNNKG	SSS	KPE	PYAGAN	RD	LFNANS	D	D		PSIQSRGLF
A/Swine/Thailand/CU33613/2023	1A.1.2	PS	KKGNS	PKLSKS	TSNDQQVLYQNA	YNNKG	SSS	KPE	PYAGAN	RD	LFNTNS	D	D		PSIQSRGLF
A/Swine/Thailand/CU33622/2023	1A.1.2	PS	KKGNS	PKLSKS	TSNDQQVLYQNA	YNNKG	SSS	KPE	PYAGAN	RD	LFNTNS	D	D		PSIQSRGLF
A/Swine/Thailand/CU33637/2023	1A.1.2	PS	KKGNS	PKLSKS	TSNDQQVLYQNA	YNNKG	SSS	KPE	PYAGAN	RD	LFNTNS	D	D		PSIQSRGLF
A/Swine/Thailand/CU33552/2023	1C	PE	KKG‐S	PKINKS	NYSDQQTLYQNN	TNDKG	SSK	TPE	SHSGAP	RE	LLTANS	D	E		PSIQSRGLF
A/Swine/Thailand/CU33558/2023	1C	PE	KKG‐S	PKINKS	NYSDQQTLYQNN	TNDKG	SSK	TPE	SHSGAP	RE	LLTANS	D	E		PSIQSRGLF
A/Swine/Thailand/CU33569/2023	1C	PE	KKG‐S	PKINKS	NYSDQQTLYQNN	TNDKG	SSK	TPE	SHSGAP	RE	LLTANS	D	E		PSIQSRGLF
A/swine/Thailand/CU35124/2024	1C	PN	KKGNS	PKLRKS	TDSDQQTLYQNN	TNNKG	SSK	TPE	SHSGAN	RE	LLTADS	D	E		PSIQSRGLF

^∗^H3 numbering.

**Table 8:** Genetic analysis of the *H3* gene of Thai‐swIAVs in this study.

Virus	HA cluster	Antigenic site							Receptor binding site	
		A	B		C	D	E			
		140–146 ^∗^	156–161 ^∗^	189–199 ^∗^	277–282 ^∗^	205–221 ^∗^	171–175 ^∗^	243–249 ^∗^	226 ^∗^	228 ^∗^
A/Wuhan/359/1995	Human	KRGSVKS	KLEYKY	SDQTSIYVQAS	CNSECI	STKRSQQTVIPNIGSRP	NDKFD	LLINSYG	I	S
A/swine/Chachoengsao/2003	EA	KRGPDSG	KSGTTY	REQTNLYVQAS	CSSECI	STKRSQQTISPNIGPRP	SDNFN	LIINSNG	L	S
A/swine/UdonThani/NIAH464/2004	EA	KRGPDSG	KLGNTY	REQTNLYVQAS	CSSECI	STKISQQTISPNIGPRP	SNNFN	LIINSNG	L	S
A/swine/Ratchaburi/NIAH59/2004	HU	KRGSVKS	KLDYKY	NDQTNLYVQAS	CNSECI	STKRSQQTVIPNIGSRP	NDKFD	LLINSTG	I	S
A/swine/Nakhonpathom/NIAH586–1/2005	EA	KRGSDNS	KSGNTY	QEQTRLYAQES	CISECI	STKRNQQTVTPNVGPRP	NDNFD	LLINSNG	L	S
A/swine/Thailand/KU5.1/2004	HUb	KRGSVKS	KLDYKY	SDQTNLYVQAS	CNSECI	STKRSQQTVIPNIGSRP	NDKFD	LLINSTG	I	S
A/swine/Chonburi/NIAH106952–026/2011	HU	KRGSIKS	KLEYKY	SDQxSLYVQAS	CNSECI	STKRGQQTVIPNIGSRP	NEKFD	LLINSTG	I	S
A/Swine/Thailand/CU‐S3689/13	HUb	KRGSVKS	KLDYKY	NNQTNLYVQAS	CNYGCI	STKRSQQTVIPNIGSRP	NDKFN	LLINSTG	I	S
A/Swine/Thailand/CU‐S14129/13	HUa	KRGYVNS	QSGHKY	SDQTSLYVQAS	CNSECV	STKRSQQTVIPNIGSRP	NEKFD	LLINSTG	I	S
A/Swine/Thailand/CU‐S14252/13	HUb	KRGSVKS	KLDYKY	SDQTNLYVQAS	CNSECI	STKRSQQTVIPNIGFRP	NDKFD	LLINSTG	I	S
A/swine/Thailand/CU3790/2017	HUb	KRGSVKS	QLNYKY	SDQTNLYVQAS	CNSECI	STKRSQQTVIPNIGFRP	NDKFD	LLINSTG	I	S
A/swine/Thailand/CU20226/2018	HUb	KRGSVKS	QLNYKY	SDQTNLYVQAS	CNSECI	STKRSQQTVIPNIGFRP	NDKFD	LLINSTG	I	S
A/swine/Thailand/CU22337/2018	HUa	KRGYVNS	HSGHKY	SDQTSLYVQTS	CNSECV	STQRSQQTVIPNIGSRP	NEKFD	LLINSTG	I	S
A/swine/Ratchaburi/NIAH874/2005	HUb	KRGSVKS	KLNYKY	SDQTNLYVQAS	CNSECI	STKRSQQTVIPNIGSRP	NDKFD	LLINSTG	I	S
A/New York/563/1996	HU	KRRSVKS	KLEYKY	SDQTSLYVQAS	CNSECI	STKRSQQTVIPNIGSRP	NDKFD	LLINSTG	V	S
This study										
A/Swine/Thailand/CU24251/2019	HUa	KRGYVNS	HSGHKY	SDQTSLYVQAS	CNSECV	STQRSQQTVIPNIGSRP	NEKFD	LLINSTG	I	S
A/Swine/Thailand/CU24271/2019	HUa	KRGYVNS	HSGHKY	SDQTSLYVQAS	CNSECV	STQRSQQTVIPNIGSRP	NEKFD	LLINSTG	I	S
A/Swine/Thailand/CU24272/2019	HUa	KRGYVNS	HSGHKY	SDQTSLYVQAS	CNSECV	STQRSQQTVIPNIGSRP	NEKFD	LLINSTG	I	S
A/Swine/Thailand/CU24274/2019	HUa	KRGYVNS	HSGHKY	SDQTSLYVQAS	CNSECV	STQRSQQTVIPNIGSRP	NEKFD	LLINSTG	I	S
A/Swine/Thailand/CU24926/2019	HUb	KRGSDKS	KLNYKY	SDQTNLYAQAS	CNSECI	STQRSYQTVIPNIGSRP	NDKFD	LLINSTG	I	S
A/Swine/Thailand/CU24929/2019	HUb	KRGSDKS	KLNYKY	SDQTNLYAQAS	CNSECI	STQRSYQTVIPNIGSRP	NDKFD	LLINSTG	I	S
A/Swine/Thailand/CU25074/2020	HUa	KRGYVNS	HSGHKY	SDQTSLYVQAS	CNSECV	STQRSQQTVIPNIGSRP	NEKFD	LLINSTG	I	S
A/Swine/Thailand/CU26133/2020	HUa	KRGYVNS	HSGHKY	SDQTSLYAQAS	CNSGCI	STQRSQQTVIPNIGYRP	NEKFD	LLINSTG	I	S
A/Swine/Thailand/CU28095/2021	HU	KRGSVNS	KFEYKY	SDQTSLYAQAS	CNSECI	STRRSQQTVIPNIEPIP	NDKFD	LLIRSTG	V	S
A/Swine/Thailand/CU28100/2021	HU	KRGSVNS	KFEYKY	SDQTSLYAQAS	CNSECI	STRRSQQTVIPNIEPIP	NDKFD	LLIRSTG	V	S
A/Swine/Thailand/CU31947/2023	HUa	KRGSTNS	KLEYKY	SDQTSLYVQAS	CNSECI	STKIGQQTVIPNIGSRS	NEKFD	LFINSTG	I	S
A/Swine/Thailand/CU31994/2023	HUa	KRGSTNS	KLEYKY	SDQTRLYVQAS	CNSECI	STKIGQQTVIPNIGSRS	NEKFD	LFINSTG	I	S
A/swine/Thailand/CU36481/2025	HUa	KRGSTNS	KLDYKY	SDQTSLYVQAS	CNSECI	STKIGQQTVIPNIGSRS	NEKFD	LLINSTG	I	S

^∗^H3 numbering.

For the genetic analysis of important mutations in internal genes (Tables 9 and 10), all Thai‐swIAVs contained E627 in the *PB2* gene, indicating low virulence in mammals [45, 46]. Most Thai‐swIAVs, except CU28095 and CU28100, showed D701 in the *PB2* gene, indicating low virulence in mammals, but contained 92D in the NS gene, which was associated with increased viral virulence [47–49]. The S31N substitution in the *M2* gene was observed in all Thai‐swIAVs, suggesting viral resistance to the antiviral drug Amantadine [50] (Table 9). There were no amino acid mutations related to Oseltamivir resistance in N1 (E119V, H275Y, R293K, and N295S) and N2 (N146K, S219T, and A272V; Table 10).

**Table 9:** Genetic analysis of amino acid substitutions on *PB2*, *M*, and *NS* genes of Thai‐swIAVs.

Virus	Subtype	PB2		NS	M2
		E627K	D701N	E92D	S31N
A/swine/Thailand/CU24248/2019	H1N1	E	D	D	N
A/swine/Thailand/CU24758/2019	H1N1	E	D	D	N
A/swine/Thailand/CU24760/2019	H1N1	E	D	D	N
A/swine/Thailand/CU26137/2020	H1N1	E	D	D	N
A/swine/Thailand/CU30318/2022	H1N1	E	D	D	N
A/swine/Thailand/CU30334/2022	H1N1	E	D	D	N
A/swine/Thailand/CU30335/2022	H1N1	E	D	D	N
A/swine/Thailand/CU30359/2022	H1N1	E	D	D	N
A/swine/Thailand/CU32449/2023	H1N1	E	D	D	N
A/swine/Thailand/CU33622/2023	H1N1	E	D	D	N
A/swine/Thailand/CU33637/2023	H1N1	E	D	D	N
A/swine/Thailand/CU35124/2024	H1N1	E	N	D	N
A/swine/Thailand/CU33552/2023	H1N2	E	D	D	N
A/swine/Thailand/CU33558/2023	H1N2	E	D	D	N
A/swine/Thailand/CU33569/2023	H1N2	E	D	D	N
A/swine/Thailand/CU24251/2019	H3N2	E	D	D	N
A/swine/Thailand/CU24271/2019	H3N2	E	D	D	N
A/swine/Thailand/CU24272/2019	H3N2	E	D	D	N
A/swine/Thailand/CU24274/2019	H3N2	E	D	D	N
A/swine/Thailand/CU24926/2019	H3N2	E	D	D	N
A/swine/Thailand/CU24929/2019	H3N2	E	D	D	N
A/swine/Thailand/CU25074/2020	H3N2	E	D	D	N
A/swine/Thailand/CU26133/2020	H3N2	E	D	D	N
A/swine/Thailand/CU28095/2021	H3N2	E	N	E	N
A/swine/Thailand/CU28100/2021	H3N2	E	N	E	N
A/swine/Thailand/CU31947/2023	H3N2	E	D	D	N
A/swine/Thailand/CU31994/2023	H3N2	E	D	D	N
A/swine/Thailand/CU32453/2023	H3N2	E	D	D	N
A/swine/Thailand/CU33613/2023	H3N2	E	D	D	N
A/swine/Thailand/CU36481/2025	H3N2	E	D	D	N

**Table 10:** Genetic analysis of amino acid substitution related to oseltamivir resistance on the NA gene of Thai‐swIAVs.

Virus	Subtype	NA1			
		E119V	H275Y	R293K	N295S
A/swine/Thailand/CU24248/2019	H1N1	E	H	R	N
A/swine/Thailand/CU24758/2019	H1N1	E	H	R	N
A/swine/Thailand/CU24760/2019	H1N1	E	H	R	N
A/swine/Thailand/CU26137/2020	H1N1	E	H	R	N
A/swine/Thailand/CU30318/2022	H1N1	E	H	R	N
A/swine/Thailand/CU30334/2022	H1N1	E	H	R	N
A/swine/Thailand/CU30335/2022	H1N1	E	H	R	N
A/swine/Thailand/CU30359/2022	H1N1	E	H	R	N
A/swine/Thailand/CU32449/2023	H1N1	E	H	R	N
A/swine/Thailand/CU33622/2023	H1N1	E	H	R	N
A/swine/Thailand/CU33637/2023	H1N1	E	H	R	N
A/swine/Thailand/CU35124/2024	H1N1	E	H	R	N

		NA2			
		N146K	S219T	A272V	245–248 deletion
A/swine/Thailand/CU33552/2023	H1N2	N	S	A	No
A/swine/Thailand/CU33558/2023	H1N2	N	S	A	No
A/swine/Thailand/CU33569/2023	H1N2	N	S	A	No
A/swine/Thailand/CU24251/2019	H3N2	N	S	A	No
A/swine/Thailand/CU24271/2019	H3N2	N	S	A	No
A/swine/Thailand/CU24272/2019	H3N2	N	S	A	No
A/swine/Thailand/CU24274/2019	H3N2	N	S	A	No
A/swine/Thailand/CU24926/2019	H3N2	N	S	A	No
A/swine/Thailand/CU24929/2019	H3N2	N	S	A	No
A/swine/Thailand/CU25074/2020	H3N2	N	S	A	No
A/swine/Thailand/CU26133/2020	H3N2	N	S	A	No
A/swine/Thailand/CU28095/2021	H3N2	N	S	A	No
A/swine/Thailand/CU28100/2021	H3N2	N	S	A	No
A/swine/Thailand/CU31947/2023	H3N2	N	S	A	No
A/swine/Thailand/CU31994/2023	H3N2	N	S	A	No
A/swine/Thailand/CU32453/2023	H3N2	N	S	A	No
A/swine/Thailand/CU33613/2023	H3N2	N	S	A	No
A/swine/Thailand/CU36481/2025	H3N2	N	S	A	No

## 4. Discussion

This multiyear swine IAV active surveillance conducted across Thailand from 2019 to 2025 provided critical insights into the current epidemiological perspectives and the evolutionary dynamics of swIAVs in the Thai pig population. Our study successfully characterized 30 Thai‐swIAVs, identifying three major circulating subtypes, swIAV‐H1N1, swIAV‐H1N2, and swIAV‐H3N2, which collectively demonstrated significant genetic reassortment, including the emergence of novel genotypes in Thailand.

### 4.1. Epidemiological Trends and Risk Factors for Thai‐SwIAVs

A multiyear swIAV surveillance showed an overall swIAV prevalence of 10.87% (177/1629). This swIAV positivity (10.87%) was higher than that in previous swIAV surveillance studies in Thailand, 6.05% in 2009–2011 [35], 1.75% in 2010–2012 [36], 6.66% in 2011–2014 [19], and 4.5% in 2011–2017 [51]. In contrast, it was lower than the swIAV prevalence in a longitudinal survey conducted on a pig farm in Thailand from 2017 to 2018 (18.81%) [38]. However, a previous longitudinal study targeted sample collection by specific pig ages (piglets and weaning pigs) and selected a farm with a history of swIAV outbreaks. swIAV positivity in the study was comparable to or slightly lower than surveillance data from other regions of Southeast Asia (SEA), but it highlighted the endemic nature of swIAV in the region. For example, previous studies reported swIAV prevalence of 11% in Myanmar, 3.7% in Vietnam, 1.5% in Cambodia, and 0.47% in China [33, 37, 52–54].

The finding that pig farms with clinical respiratory signs showed a 95%–100% swIAV positivity strongly supported the usefulness of targeted surveillance based on clinical signs. Importantly, the highest swIAV positivity was in nursery pigs (15.37%), followed by suckling pigs (9.94%), confirming that swIAVs mainly affect nursery and weaning pigs [38, 55]. This pattern was typical of swIAV infections worldwide and indicated that current farm management and/or vaccination practices may not fully protect piglets during the critical weaning phase. The swIAV positivity rate was high during the rainy season (14.16%). This finding was consistent with a previous report indicating that the prevalence of swIAVs is low in summer and high in both rainy and winter seasons [56]. It is noted that a humid environment supports virus survival and increases the chance of contact transmission. Additionally, the disproportionately high prevalence observed in some provinces, for example, Suphanburi (47.50%) and Ratchaburi (19.27%), suggested local risk factors, possibly related to pig density, trade movement, or specific biosecurity gaps in particular pig farms. These pig farms in hotspots required routine investigation to identify and mitigate the transmission risks posed by swIAVs. This study identified 3 subtypes of swIAVs (swIAV‐H1N1, swIAV‐H1N2, and swIAV‐H3N2) among 30 isolates. The swIAV subtype proportion was 46.67% (14/30) of swIAV‐H1N1, 10.00% (3/30) of swIAV‐H1N2, and 43.33% (13/30) of swIAV‐H3N2. The proportion of swIAV subtypes was consistent with previous studies, indicating that swIAV‐H1N1 and swIAV‐H3N2 were the predominant subtypes and that swIAV‐H1N2 was a minor, reemerging subtype in Thailand [19, 35, 36, 38].

### 4.2. Phylogenetic Relationships of Thai‐SwIAVs

Phylogenetic analysis of H1 showed that Thai swIAV‐H1 belonged to the CS, sublineage 1A.1.2 (1A.1.2;*n* = 13), and the EA (1C; *n* = 4). After pdmH1N1‐2009 was introduced into the Thai pig population, the H1 of pdmH1N1‐2009 (1A.3.3.2) was sometimes replaced in the Thai‐swIAV genome but was not observed in this study [19, 38]. In this study, the H1 of enH1N1 (CU35124) and swIAV‐H1N2 (CU33552, CU33558, and CU33569) was grouped into the EA. It was suggested that their ancestors originated from European swIAVs and may have been endemic to the Thai pig population for a period of time. Phylogenetic analysis of N1 showed that the *N1* gene of nine swIAV‐H1N1 belonged to EA. Similarly, enH1N1, isolated in 2024, was also grouped in the EA. While the swIAV‐H1N1, isolated in 2019 and 2020 (*n* = 4), was grouped with the pdmH1N1‐2009 group. Our results aligned with the previous studies that the *N1* gene of Thai swIAVs was either grouped with the EA or the pdmH1N1‐2009 lineages [14, 17, 19, 22, 38, 39].

Phylogenetic analysis of the *H3* and *N2* genes showed that most swIAV‐H3N2 belonged to the HU lineage. swIAV‐H3N2 was clustered in human‐like A sublineage (HUa; *n* = 9) and HUb sublineage (*n* = 2), similar to previous Thai swIAV‐H3N2 [17, 19, 38, 39, 51]. Notably, the other two swIAV‐H3N2 isolates (CU28095 and CU28100), isolated in 2021, were distinct from the previously reported Thai swIAV‐H3N2. Both were closely related to the human influenza strain isolated in 1997–1998. The origin of the *H3* and *N2* genes in both Thai swIAV‐H3N2 remains unclear. It may be missing the genetic information of the common ancestors of the two viruses.

### 4.3. Genetic Diversity and Novel Reassortant Thai‐SwIAVs

The genetic constellation is crucial for understanding the evolution of swIAVs. Our results revealed an increasingly complex genetic composition of swIAVs in Thailand, driven primarily by reassortment between endemic EA‐like and pandemic H1N1‐2009 (pdmH1N1‐2009) lineages, with some contributions from CS and HU lineages. According to genetic constellation analysis, the Thai‐swIAVs in this study comprised eight genotypes: four genotypes of swIAV‐H1N1, one genotype of swIAV‐H1N2, and three genotypes of swIAV‐H3N2. The four genotypes have been previously reported in Thailand, rH1N1 (7pdm + 1CS), rH1N1 (6pdm + 1EA + 1CS), rH1N2 (6pdm + 1EA + 1HU), and rH3N2 (6pdm + 2HU), indicating that these genotypes are continuously circulating in the Thai pig population for several years [19, 35, 38, 39]. rH1N2 (6pdm + 1EA + 1HU), isolated in 2023, is particularly notable, as it had not been officially reported in Thailand in the FluDB or GISAID databases since 2013. This reemergence highlighted the need for continuous, broad surveillance as previously dormant or undetected swIAVs can reestablish circulation. The rH3N2 (6pdm + 2HU), which replaced older endemic strains, clearly demonstrated the successful establishment of the pdmH1N1‐2009 internal gene cassette as the dominant backbone in Thai swIAV‐H3N2 [19, 38]. This genotype has demonstrated the evolutionary fitness of the virus, persisting from 2019 to 2025.

The other four genotypes have never been reported in Thailand. Two new genotypes, rH1N1 (5pdm + 2EA + 1CS) and rH3N2 (4pdm + 2CS + 2HU), were reassortant viruses derived from endemic Thai swIAVs and pdmH1N1‐2009. The rH1N1 (5pdm + 2EA + 1CS) was recovered in 2022, with the pdmH1N1‐2009 backbone and the acquisition of two surface genes and one internal gene from the EA and CS. This pattern suggested continuous gene flow from the EA (en‐swIAV reservoir), a pattern consistent with global swIAV evolution, in which the EA is frequently integrated into circulating swine strains [28]. The rH3N2 genotype (4pdm + 2CS + 2HU), isolated in 2019, has a highly mosaic genome, incorporating genes from three distinct origins (pdm, CS, and HU). Such complex reassortment events are characteristic of the “mixing vessel” environment of the pig, increasing the likelihood of generating strains with enhanced transmissibility or an altered host range [2]. One genotype, enH3N2 (6EA + 2HU), was a reassortant virus between endemic swIAVs and human influenza. The enH3N2 (6EA + 2HU), isolated in 2021, marked the recurrence of an endemic H3N2 lineage, reinforcing the dual circulation of EA‐based and pdm‐based reassortants in the Thai swine herd. Another genotype, enH1N1 (8EA), was closely related to European swIAVs. The detection of a nonreassortant swIAV‐H1N1 that is composed entirely of EA‐lineage genes in 2024 is important. This enH1N1 (8EA) is closely related to swIAVs isolated in Denmark in 2020 and 2021. This suggested a direct introduction of an entire European EA‐swIAV into the Thai pig population via potentially international animal or semen trade or long‐distance migratory bird transmission. Establishing this pure EA lineage could complicate vaccine selection as current vaccines may primarily target pdmH1N1‐2009. It should be noted that a limitation of this study was that antigenic characterization by the HI test was not performed for the novel reassortant viruses identified. Thus, the HI test for cross‐reactivity and animal experiments for the virulence test should be investigated in the future.

It is known that IAV infection in pigs, can lead to the emergence of novel influenza viruses through genetic reassortment. Some novel reassortant viruses contain genes of different origins (avian, swine, and human IAVs) and can be virulent, e.g., pandemic H1N1‐2009 and H1N1‐G4. For example, after the H1N1‐2009 pandemic, the virus was isolated from pigs in many countries. Thus, several reassorted swIAVs with genes from the pandemic H1N1‐2009 virus, TR, and endemic swIAVs have been reported. In this study, the novel Thai swIAV genotypes did not have a genetic composition identical to the previously reported reassorted virus in Cambodia, China, Myanmar, South Korea, and Vietnam [37, 57–60].

Note that in a previous study, one of the important reassortant swIAVs was swIAV‐H1N1 genotype *G4* (5pdm + 2EA + 1TR), which was identified in China. The *H1N1-G4* genotype contained H1 and N1 from *EA, PB2*, *PB1*, *PA*, and *M* genes from pdmH1N1‐2009 and the *NS* gene from the triple‐reassortant (TR). In an experimental setting, H1N1‐G4 can bind to mammalian receptors, causing high levels of viral infection in human respiratory epithelial cells. In vivo, the virus can infect and transmit in ferrets [28, 61]. The H1N1‐G4 virus was predominant in the pig population in China and can be transmitted from pigs to humans, with 10.4% of human cases infected with G4 viruses and two human case reports of H1N1‐G4 virus infection leading to severe infection and even death [61]. Although novel Thai swIAV genotypes were unrelated to H1N1‐G4 in China, the virus indicated that new reassortant swIAVs with other gene patterns from pdmH1N1‐2009 and endemic swIAVs may be capable of infecting humans and potentially causing a pandemic.

### 4.4. Molecular Makers of Thai‐SwIAVs

Analysis of key amino acid substitutions provided insights into the potential virulence and zoonotic capacity of Thai‐swIAVs. The analysis of H1 receptor binding showed two swIAV‐H1N1 (CU24248 and CU24758) with the 190D and 225G, crucial molecular signatures suggesting preferential binding to both avian (alpha 2,3) and mammalian (alpha 2,6) receptors [47]. This binding capacity is considered a prerequisite for efficient zoonotic transmission and adaptation to humans. For the H3 receptor binding, all swIAV‐H3N2 exhibited 226I/V and 228S, suggesting preferential binding to the mammalian receptor. Coupled with the fact that these *H3N2* possess *H3* and *N2* genes derived from the HU lineage, this subtype presented a persistent and high‐level risk of zoonotic spillover to occupational workers [62]. All Thai‐swIAVs retained the PB2 substitution E627, generally associated with low virulence in mammals [48]. However, the co‐occurrence of 701N in PB2 and 92D in NS in some isolates suggested an adaptive pathway that may increase virulence or replication efficiency in the swine host [63]. Regarding drug resistance, the universal presence of the S31N substitution in the *M2* gene across all 30 Thai‐swIAVs confirmed widespread resistance to Amantadine and Rimantadine. This finding is consistent with global reports on the circulation of pdmH1N1‐derived internal genes and indicates that Amantadine is ineffective for treating swIAV [64, 65]. On the other hand, Oseltamivir (Tamiflu) remained the primary antiviral treatment option. The amino acid substitution conferring oseltamivir resistance in the *NA1* and *NA2* genes was not observed in Thai swIAVs. This result suggested that all swIAVs in this study were sensitive to oseltamivir [66].

### 4.5. Public Health Implications and One Health Strategies

In conclusion, the 2019–2025 swIAV active surveillance in Thailand revealed a highly dynamic, reassortment‐prone viral ecosystem defined by the cocirculation of three swIAV subtypes and the emergence of multiple novel genotypes. These findings highlighted the critical role of the Thai swine population as a significant “mixing vessel” for influenza viruses, posing a threat to both animal and human health. The most significant public health concern arose from the circulation of swIAVs with mammalian‐adapted receptors, particularly swIAV‐H1N1 and swIAV‐H3N2 (of HU origin). The risk of zoonotic spillover of the virus is primarily among high‐exposure occupations, such as pig farm workers, veterinarians, and slaughterhouse employees [28, 67]. Addressing this complex swIAV threat requires a coordinated, multisector approach under the One Health framework [68]. An integrated One Health surveillance system should combine data from veterinary and public health sources. Recommendations regarding the restricted movement of pigs, routine vaccination among workers, and the interfaces between farm workers and pigs require action to improve farm biosecurity.

## Author Contributions

Supassama Chaiyawong drafted the manuscript. Supassama Chaiyawong, Hnin Wai Phyu, and Chanakarn Nasamran performed sample collection, virus detection, and virus isolation. Supassama Chaiyawong, Hnin Wai Phyu, Chanakarn Nasamran, and Kamonpan Charoenkul performed virus characterization and phylogenetic analysis. Supassama Chaiyawong, Chanakarn Nasamran, and Kamonpan Charoenkul participated in the statistical analysis. Supassama Chaiyawong, Kamonpan Charoenkul, Somsak Pakpinyo, and Alongkorn Amonsin designed the study, performed data analysis, drafted, revised, and approved the manuscript.

## Funding

The C2F program of Chulalongkorn University supports the first author’s PostDoc fellowship. This study was supported by the Thailand Science Research and Innovation Fund, Chulalongkorn University, FF69 (Grant HEA_FF69_076_3100_006). Chulalongkorn University also provided financial support to the Center of Excellence for Emerging and Re‐emerging Infectious Diseases in Animals (CUEIDAs).

## Disclosure

All the authors reviewed the manuscript.

## Ethics Statement

This study was conducted under the Chulalongkorn University Animal Care and Use Committee (CU‐ACUC) under protocol number CU‐VET IACUC# 2131033. This research followed the ARRIVE guidelines.

## Consent

Prior to sample collection, verbal informed consent was obtained from all participating farm owners.

## Conflicts of Interest

The authors declare no conflicts of interest.

## Supporting Information

Additional supporting information can be found online in the Supporting Information section.

## Supporting information


**Supporting Information 1** Figure S1: Pig density distribution by province in Thailand (2024). Figures S2–S7: Phylogenetic tree of internal gene segments of Thai swIAV (PB2, PB1, PA, NP, M, and NS). The phylogenetic tree was generated using the neighbor‐joining algorithm with 1000 bootstrap replicates in MEGA12.0. The black circle represents swIAV characterized in this study.

**Supporting Information 2** Table S1. Influenza A virus detection and viral isolation by egg inoculation and cell culture in this study.

## Data Availability

The data that support the findings of this study are openly available in the GenBank database at https://www.ncbi.nlm.nih.gov/genbank/ with Reference Numbers PX444173–PX444412.
