# Supplementary material for: Genetic Characterization of Swine Influenza Viruses in Thailand in 2019–2025 Reveals Novel Reassortants
Source: Transbound Emerg Dis. 2026 Jun 2;2026:9516354. doi: 10.1155/tbed/9516354 (PMC13239211; doi:10.1155/tbed/9516354)

**Supplement Figure 1.** Pig density distribution by province in Thailand (2024)


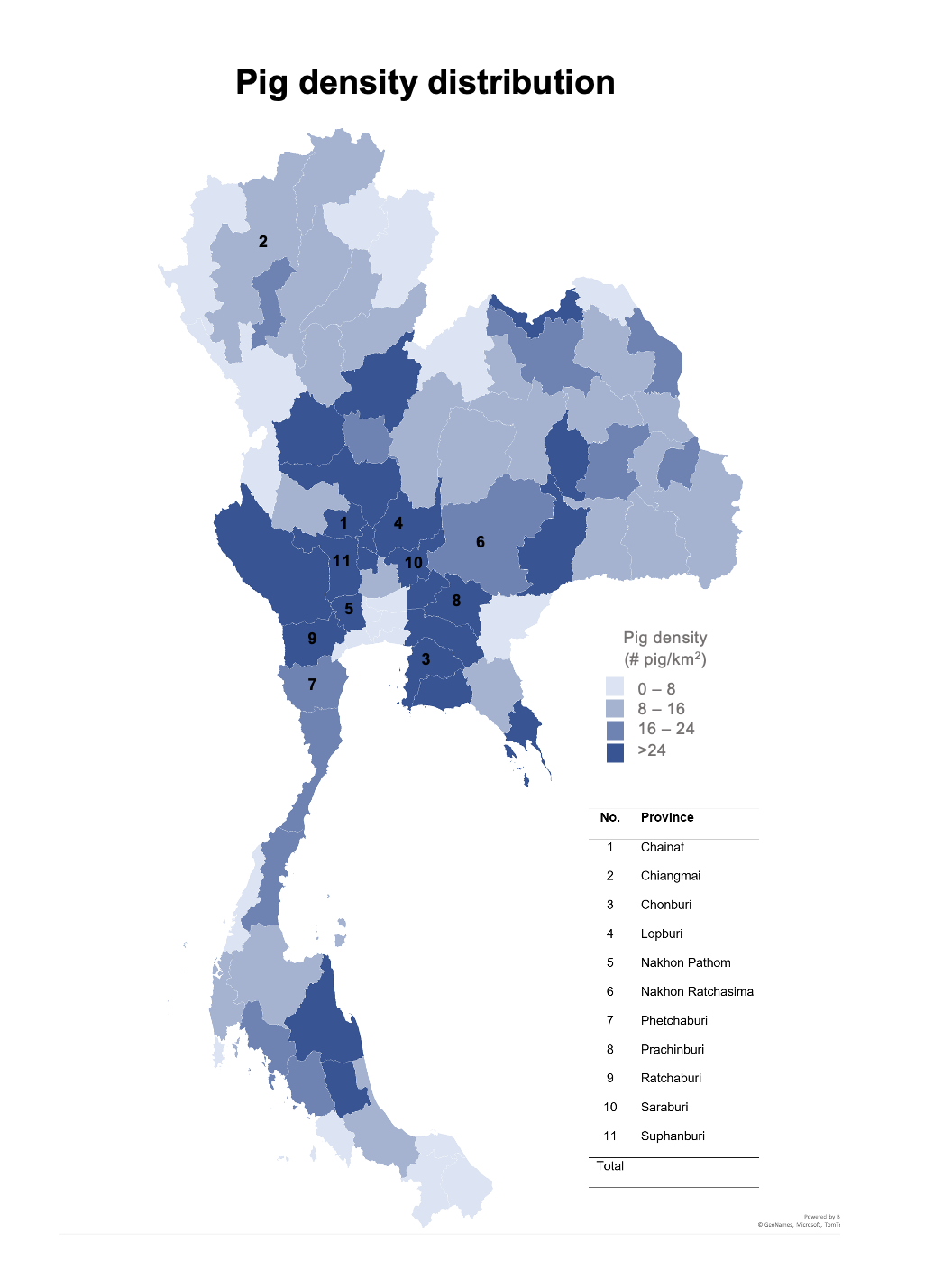


**Supplement Figure 2-7.** Phylogenetic tree of internal gene segments of Thai swIAV (PB2, PB1, PA, NP, M, and NS). The phylogenetic tree was generated using the neighbor-joining algorithm with 1,000 bootstrap replicates in MEGA12.0. The black circle represents swIAV characterized in this study.

**PB2**


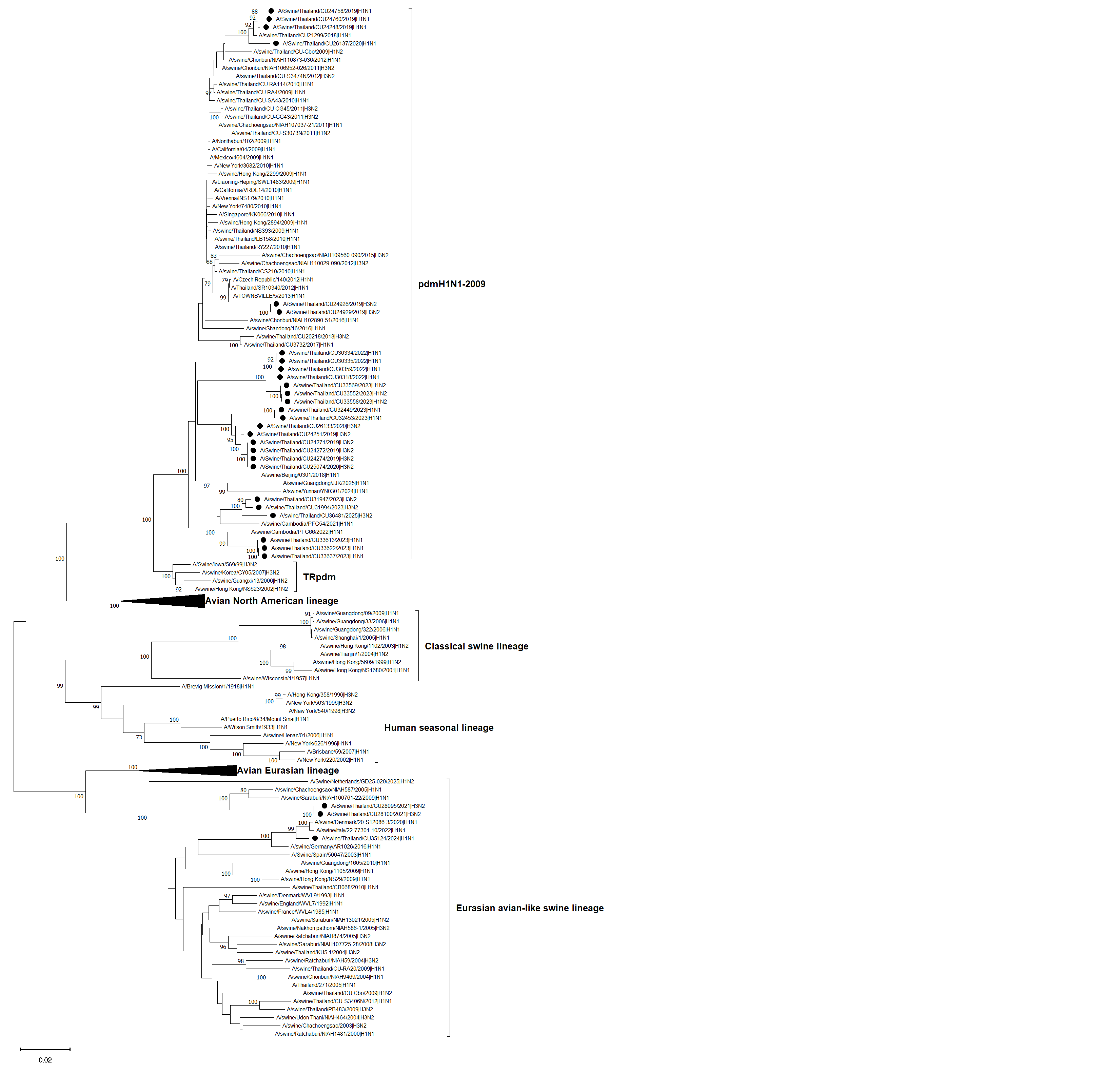


**PB1**


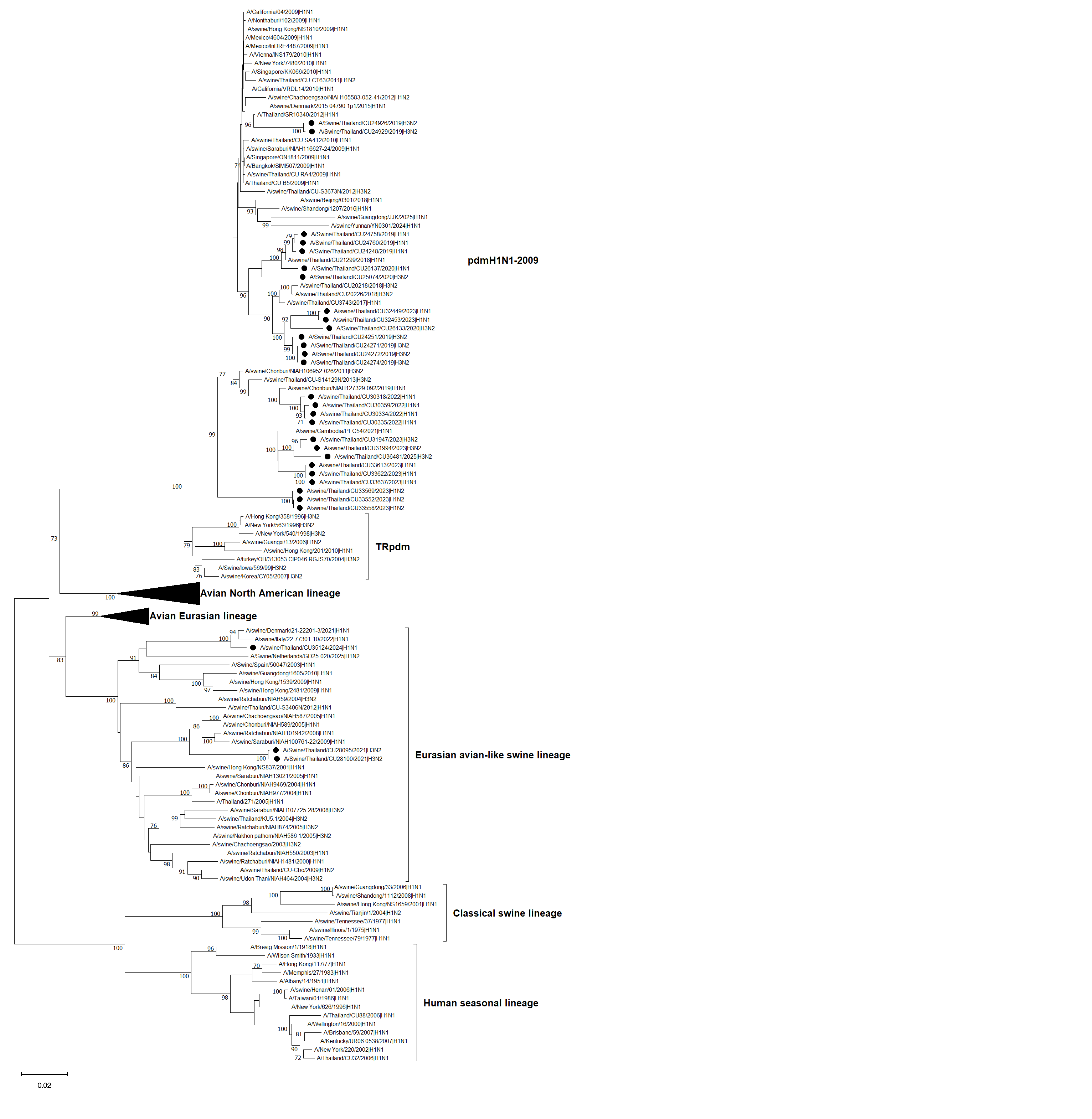


**PA**


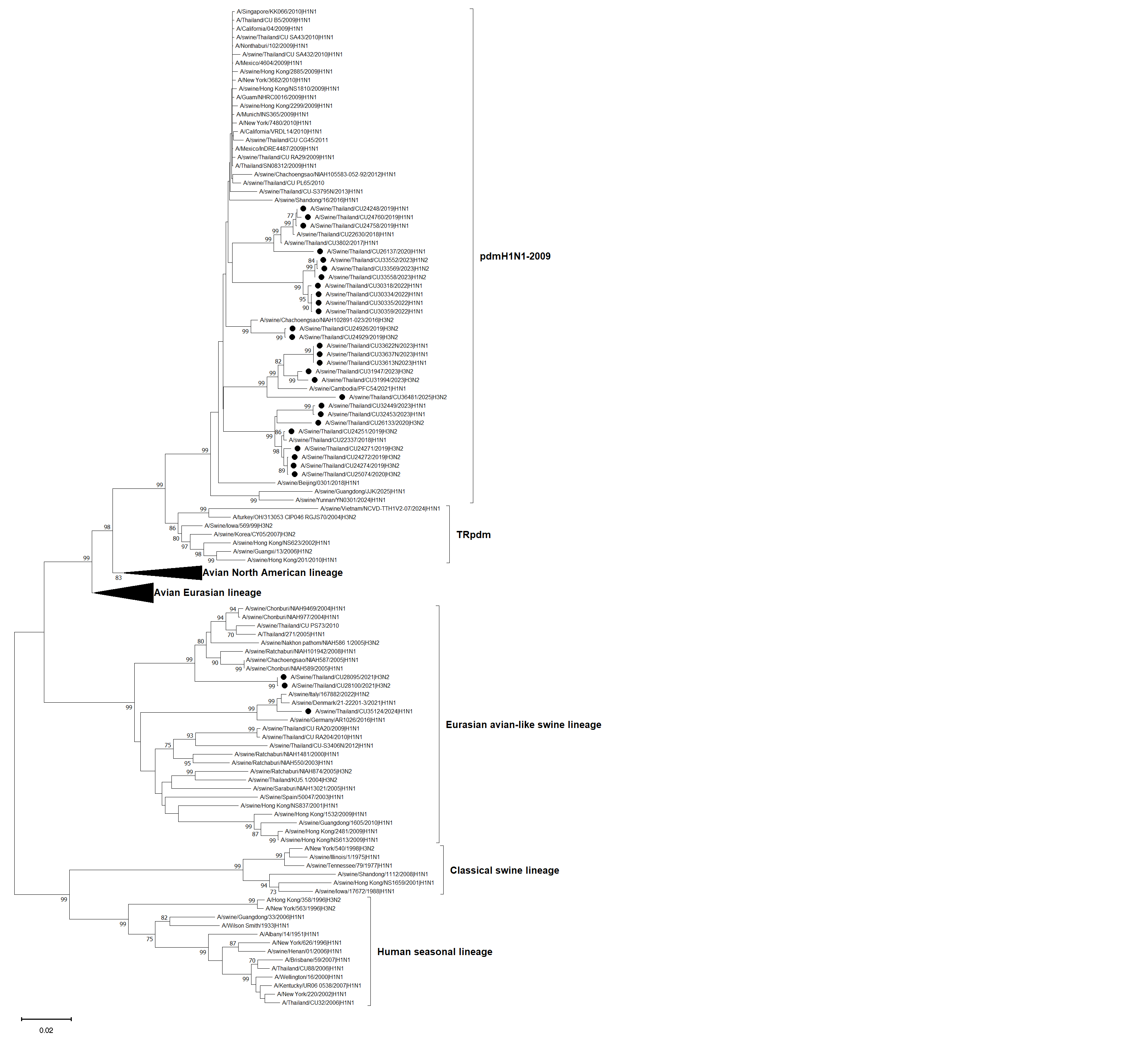


**NP**


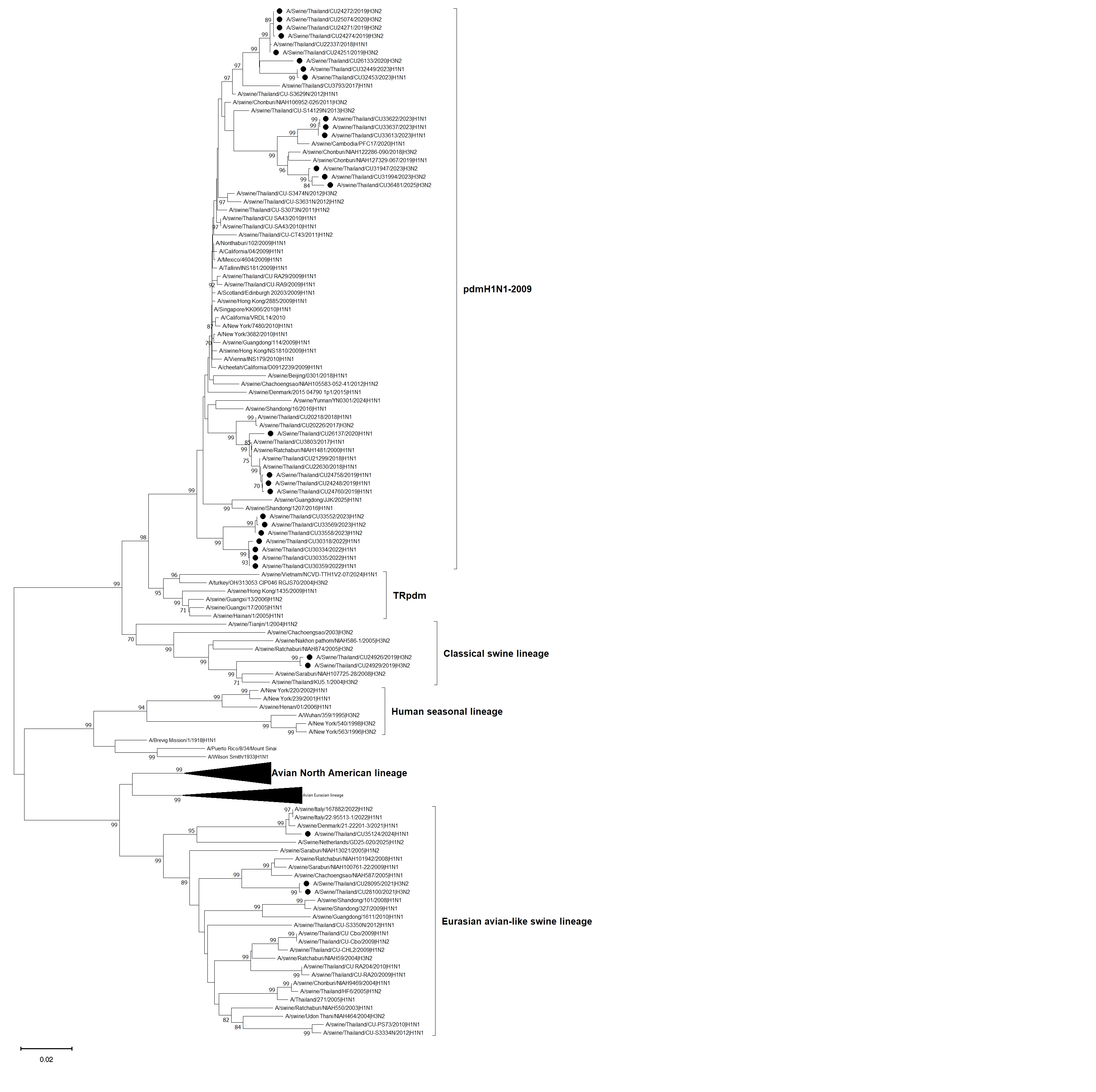


**M**


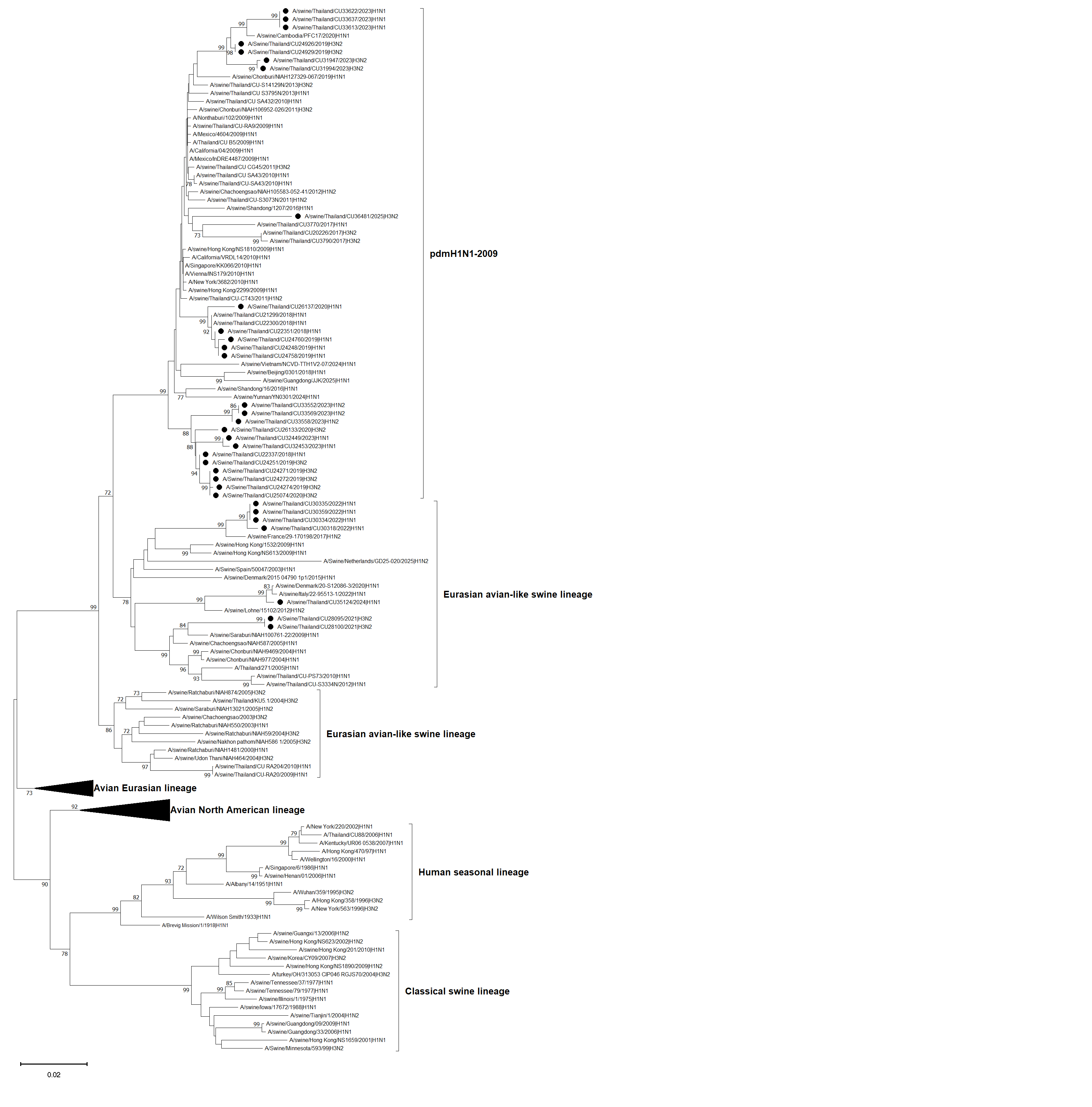


**NS**


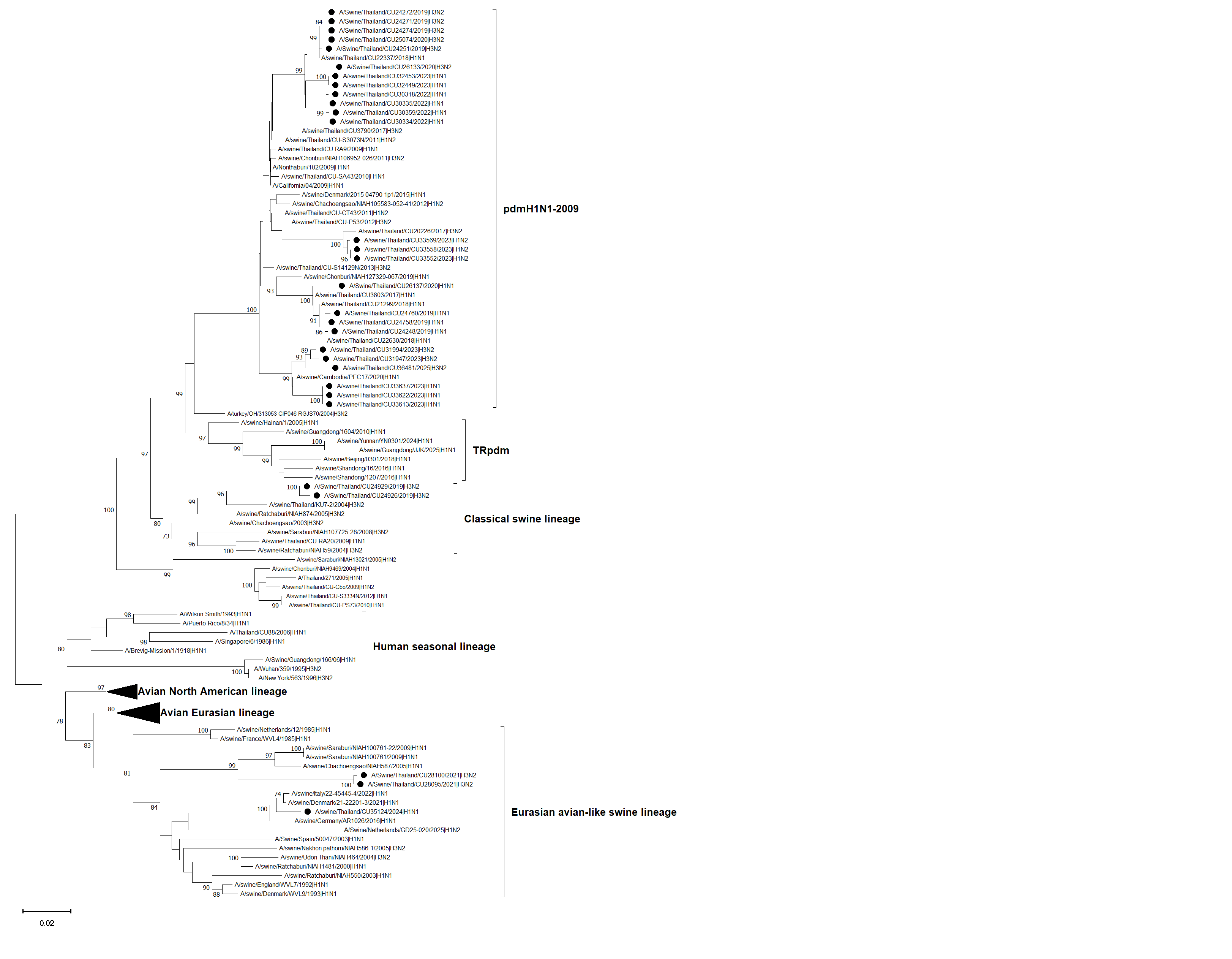

Supplement: Supplementary file 1 — Supporting Information 1 Figure S1: Pig density distribution by province in Thailand (2024). Figures S2–S7: Phylogenetic tree of internal gene segments of Thai swIAV (PB2, PB1, PA, NP, M, and NS). The phylogenetic tree was generated using the neighbor‐joining algorithm with 1000 bootstrap replicates in MEGA12.0. The black circle represents swIAV characterized in this study. [file TBED-2026-9516354-s002.docx]
