# Supplementary material for: Genetic Characterization of Swine Influenza Viruses in Thailand in 2019–2025 Reveals Novel Reassortants
Source: Transbound Emerg Dis. 2026 Jun 2;2026:9516354. doi: 10.1155/tbed/9516354 (PMC13239211; doi:10.1155/tbed/9516354)
Supplement: Supplementary file 2 — Supporting Information 2 Table S1. Influenza A virus detection and viral isolation by egg inoculation and cell culture in this study. [file TBED-2026-9516354-s001.docx]

**Supplement Table 1.** Influenza A virus detection and viral isolation by egg inoculation and cell culture in this study.

| Year | Sample collection | IAV detection  (Realtime-RT PCR) | | IAV isolation  (Egg inoculation) | | IAV isolation  (Cell culture) | |
| --- | --- | --- | --- | --- | --- | --- | --- |
|  | # sample | # sample tested | # positive (suspected) | # sample tested | # positive (%) | # sample tested | # positive (%) |
|  |  |  |  |  |  |  |  |
| 2019 | 123 | 123 | 20(8) | 28 | 3 | 25 | 6 |
| 2020 | 80 | 80 | 7(1) | 8 | 2 | 6 | 1 |
| 2021 | 8 | 8 | 3(1) | 4 | 1 | 3 | 1 |
| 2022 | 542 | 542 | 11(7) | 18 | 4 | 14 | - |
| 2023 | 526 | 526 | 120(21) | 141 | 10 | 131 | - |
| 2024 | 200 | 200 | 6(8) | 14 | 1 | 13 | - |
| 2025 | 150 | 150 | 10(3) | 13 | 1 | 12 | - |
|  |  |  |  |  |  |  |  |
| Total | 1,629 | 1,629 | 177(49) | 226 | 22 (9.73%)* | 204 | 8 (3.92%)** |

*Isolation rate for Egg inoculation assay: 22/226 (9.73%)

** Isolation rate for Cell culture assay: 8/204 (3.92%)
